# Identification by Genome Mining of a Type I Polyketide Gene Cluster from *Streptomyces argillaceus* Involved in the Biosynthesis of Pyridine and Piperidine Alkaloids Argimycins P

**DOI:** 10.3389/fmicb.2017.00194

**Published:** 2017-02-10

**Authors:** Suhui Ye, Brian Molloy, Alfredo F. Braña, Daniel Zabala, Carlos Olano, Jesús Cortés, Francisco Morís, José A. Salas, Carmen Méndez

**Affiliations:** ^1^Departamento de Biología Funcional e Instituto Universitario de Oncología del Principado de Asturias, Universidad de OviedoOviedo, Spain; ^2^EntreChem, S.LOviedo, Spain

**Keywords:** cryptic, type I polyketide synthase, pyridine, piperidine, thioester reductase, growth, alkaloid, *Streptomyces*

## Abstract

Genome mining of the mithramycin producer *Streptomyces argillaceus* ATCC 12956 revealed 31 gene clusters for the biosynthesis of secondary metabolites, and allowed to predict the encoded products for 11 of these clusters. Cluster 18 (renamed cluster *arp*) corresponded to a type I polyketide gene cluster related to the previously described coelimycin P1 and streptazone gene clusters. The *arp* cluster consists of fourteen genes, including genes coding for putative regulatory proteins (a SARP-like transcriptional activator and a TetR-like transcriptional repressor), genes coding for structural proteins (three PKSs, one aminotransferase, two dehydrogenases, two cyclases, one imine reductase, a type II thioesterase, and a flavin reductase), and one gene coding for a hypothetical protein. Identification of encoded compounds by this cluster was achieved by combining several strategies: (i) inactivation of the type I PKS gene *arpPIII*; (ii) inactivation of the putative TetR-transcriptional repressor *arpRII*; (iii) cultivation of strains in different production media; and (iv) using engineered strains with higher intracellular concentration of malonyl-CoA. This has allowed identifying six new alkaloid compounds named argimycins P, which were purified and structurally characterized by mass spectrometry and nuclear magnetic resonance spectroscopy. Some argimycins P showed a piperidine ring with a polyene side chain (argimycin PIX); others contain also a fused five-membered ring (argimycins PIV-PVI). Argimycins PI-PII showed a pyridine ring instead, and an additional *N*-acetylcysteinyl moiety. These compounds seem to play a negative role in growth and colony differentiation in *S. argillaceus*, and some of them show weak antibiotic activity. A pathway for the biosynthesis of argimycins P is proposed, based on the analysis of proposed enzyme functions and on the structure of compounds encoded by the *arp* cluster.

## Introduction

Natural products have been a productive source of bioactive compounds and drugs. By the mid-1990s, >50% of all new drugs were either natural products or analogs (Cragg et al., 1997). This frequency has decreased since then, due to the increased efforts in synthetic small molecules, while the isolation and characterization of new natural products from crude extracts remains perceived as time-consuming and labor-intensive. However, natural products and their analogs still account for >40% of small molecule drugs under development (Newman and Cragg, 2016). The importance of drug development based on natural products and their analogs is, in part, associated with their structural diversity, which facilitates opening up unexplored chemical spaces (Bauer et al., 2010). The growing appreciation of functional assays and phenotypic screens may further contribute to a revival of interest in natural products for drug discovery (Harvey et al., 2015).

Streptomycetes are Gram-positive bacteria with high GC DNA content that are mainly found in terrestrial and aquatic environments, and show a complex development cycle, involving formation of substrate and aerial mycelium, and spores (Flärdh and Buttner, 2009; Barka et al., 2016). Moreover, they are one of the most important sources of microbial bioactive compounds. These compounds are secondary metabolites that show, among others, antibiotic, antifungal, antiparasitic, immunosuppressive or herbicide activities. In addition, some of them also play a role in colony development in these microorganisms (Barka et al., 2016). Most bioactive compounds belong to the polyketide, peptide, hybrid polyketide-peptide and terpene groups. Polyketides (PK) are synthesized by macroenzyme complexes called polyketide synthases (PKS) through the condensation of small acyl-CoA units. Three different types of PKS have been described so far. Type I or modular PKS are typically organized into modules, each one responsible for a single elongation cycle. Each module contains a β-ketoacyl synthase (KS), an acyltransferase (AT) and an acyl carrier protein (ACP) domain. AT domains select the appropriate acyl-CoA unit that will be used in the corresponding extension cycle; KS domains are responsible for the decarboxylative Claisen condensations of acyl-CoA units; ACPs are non-catalytic domains that tether the growing PK chain and building block on its phosphopantetheine arm. After every condensation step, the resulting β-keto group of the biosynthesis intermediate could be modified before the next elongation step by functional domains optionally present in the corresponding module: ketoreductase (KR), dehydratase (DH), and enoyl reductase (ER). KRs stereospecifically reduce the carbonyl group of the β-ketoacyl-ACP intermediates leading to the formation of a β-hydroxyl group; DH domains catalyze reversible dehydration events to yield double bond formation between the α- and β-carbons in the polyketide chain; ER domains catalyze the reduction of double bonds to form fully reduced methylene in the carbon chain (Hertweck, 2009; Weissman, 2015).

Novel compounds are still needed to address uncovered medical needs, like infectious diseases, or cancer. Natural products possess desirable structural and chemical properties that make suitable as potent drugs, but steep technological challenges associated with screening and manufacturing of these molecules has stifled the discovery and development of natural products. The recent development of genomics, metagenomics and high-throughput screening has increased exponentially the volume of useful genetic sequence information that can be employed for natural products discovery. Additionally, a new manufacturing paradigm employing metabolic engineering and synthetic biology as its engine has greatly accelerated the path of development for microbial natural product drugs (Rutledge and Challis, 2015; Katz and Baltz, 2016; Pawar et al., 2016). Genome mining consists of searching a genome for genes that encode enzymes involved in a particular process. In recent years, genome mining has been applied to streptomycetes and has become a new and quick approach to identify previously unknown gene clusters (Olano et al., 2014; Rutledge and Challis, 2015; Ochi, 2016). In most cases these clusters are silent or lowly expressed under laboratory conditions. Therefore, several strategies have been developed to awake and/or increase their expression, and to identify the compounds encoded by these gene clusters, which involve both the use of genetic engineering approaches and the use of different media and culture conditions (Olano et al., 2014; Rutledge and Challis, 2015; Ochi, 2016).

In this paper we report the genome mining of *Streptomyces argillaceus* ATCC 12956, a producer of the known antitumoral drug mithramycin, and the use of this approach to uncover cryptic pathways, lowly or not expressed by the wild type strain. One of these pathways lowly expressed under standard laboratory conditions, has been overexpressed and the products isolated and characterized chemically and biologically, namely the argimycin P family of compounds.

## Materials and Methods

### Bacterial Strains, Culture Conditions, Plasmids and DNA Manipulations

*Streptomyces argillaceus* ATCC 12956, and *S. argillaceus* GIH, *S. argillaceus* AFTA and *S. argillaceus* AFTA-GIH (Zabala et al., 2013) were used as source of DNA and for gene replacement and expression experiments, and/or production of argimycins P. For sporulation the strains were grown for 7 days at 30°C on agar plates containing medium A (Fernández et al., 1998). *Streptomyces* sp. NRRL S-1022 was used to test argimycins P production. SM10 and SM17 media were used for argimycins P production by *S. argillaceus* and *Streptomyces* S-1022, respectively. When required, antibiotics were added to media at the following final concentrations: ampicillin (100 μg/mL), kanamycin (50 μg/mL), nalidixic acid (25 μg/mL), apramycin (25 μg/mL), and thiostrepton (50 μg/mL). A pKC505-based cosmid library of *S. argillaceus* genome DNA was used to identify cosmids containing *arp* genes (Lombó et al., 1996). *Escherichia coli* DH10B (Invitrogen) and *E. coli* ET12567/pUB307 (Kieser et al., 2000) were used as cloning hosts for plasmid propagation and for conjugation experiments, respectively. Antibiotic activity of argimycins P was assayed against *Micrococcus luteus, Escherichia coli* and *Saccharomyces cerevisiae*, as described Vilches et al. (1990). Plasmids pCR-Blunt (Invitrogen) and pUO9090 (M. C. Martín, unpublished results), were used for subcloning. Plasmids pHZ1358 (Sun et al., 2002) and pBSKTT (this work) were used for generating mutants by gene replacement and gene disruption. pEM4ATC (C. Cano-Prieto, unpublished results) and pIAGO (Aguirrezabalaga et al., 2000) were used for gene expression in *S. argillaceus*. pBSKTT was constructed by cloning a PstI fragment containing the *oriT* from pEM4T (Menéndez et al., 2006), into the PstI site of pBSKT (Lombó et al., 1999). DNA manipulations, transformations and intergeneric conjugations were performed according to standard procedures for *E. coli* (Sambrook and Russell, 2001) and for *Streptomyces* (Kieser et al., 2000). Herculase (Stratagene) and 2.5% dimethyl-sulfoxide (DMSO) were used for PCR amplifications. Purified amplicons were sequenced and compared to others in databases.

### Sequencing and Analysis of DNA

*Streptomyces argillaceus* genome DNA sequence was obtained by Life Sequencing (Valencia, Spain; pyrosequencing 454 Life Science-Roche platform). Paired end sequences were *de novo* assembled using the Newbler assembler v. 2.8 (Margulies et al., 2005). Sequence of cluster 18 was completed using PCR fragments (Sanger et al., 1977) (DNA sequencing service; University of Oviedo). Annotation was performed using PGAAP (Prokaryotic Genomes Automatic Annotation Pipeline) (Angiuoli et al., 2008). Database searching and sequence analysis were carried out using antiSMASH (Antibiotic and Secondary Metabolite Analysis Shell) (Blin et al., 2013; Medema et al., 2015; Weber et al., 2015), and BLAST (Altschul et al., 1997). Analysis of domains in PKS and NRPS were carried out using ASMPKS (Tae et al., 2007) and NRPS predictor (Rausch et al., 2005). Search for transmembrane domains was carried out using the TMHMM v.2.0 (Krogh et al., 2001).

### Plasmid Constructs for Gene Expression and Generating Mutants

Several plasmids were generated as described in Supplementary Material, either to express *arp* genes or to generate mutants in *S. argillaceus* (**Table 1**). Mutants were generated by either disrupting the target gene by inserting a plasmid, or by replacing a DNA region by an apramycin resistance cassette that was inserted in the same direction of transcription. These plasmids were independently introduced by conjugation into *S. argillaceus*, and transconjugants were selected either with thiostrepton (pBSKTT-based plasmids) or with apramycin (pHZ1358-based plasmids). In the last case, apramycin-resistance, thiostrepton-sensitive colonies were selected. Mutants were confirmed by PCR amplification using specific oligonucleotides (Supplementary Table S1), and sequencing the PCR products.

**Table 1:** Mutant and recombinant *S. argillaceus* strains generated in this work.

Strains	Plasmid used	Gene(s)^∗^
**Mutant strains**		
MARPN	pHZMutAT	*arpN*
MARPPIII	pBSKTT1701	*arpPIII*
MARPRI	pHZMutSARP	*arpRI*
MARPRII	pHZMutTetR	*arpRII*
MORF3	pHZMutorf3	*orf3*
MORF4	pHZMutorf17	*orf4*
MORF9	pHZMutNAcTr	*orf9*
DARPO-HII	pHZDel59b	*arpO, arpDHI, arpDHII, arpN, arpK, arpHI, arpHII*
DORF5-7	pHZDel1820	*orf5, orf6, orf7*
DORF11-16	pHZDel13	*orf11, orf12, orf13, orf14, orf15, orf16*
DORF19-21	pHZDel2	*orf19, orf20, orf21*
**Recombinant strains**		
MARPPIII (pEM4ATCPKS)	pEM4ATCPKS	*arpPIII, arpT*
DARPO-HII (pIAGOorf8)	pIAGOorf8bis	*arpN*

*^∗^Genes mutated or expressed.*

### UPLC Analysis and Purification of Argimycins P

Culture samples (1 ml) were extracted with 1 volume of *n*-butanol. Organic extracts were dried under vacuum, and residues were dissolved in a small volume of DMSO: methanol (50:50). Analysis of argimycins P production was performed by reversed-phase chromatography on Acquity UPLC equipment with a BEH C18 column (1.7 μm, 2.1 × 100 mm; Waters, Milford, MA, USA) with acetonitrile and 0.1% trifluoroacetic acid (TFA) in water as eluent. Samples were eluted with 10% acetonitrile for 1 min, followed by a linear gradient from 10 to 61.4% acetonitrile over 4 min at a flow rate of 0.5 ml/min and a column temperature of 30°C. Detection and spectral characterization of peaks were carried out with a photodiode array detector and Empower software (Waters). Chromatograms were extracted at 400, 272, and 230 nm.

For purification purposes, *S. argillaceus* MARPRII was grown by a two-step culture method, as previously described Fernández et al. (1998). In the production step, 40 250-milliliter Erlenmeyer flasks, each containing medium (50 mL), were incubated for 3 days. The cultures were centrifuged and filtered, and applied to a solid-phase extraction cartridge (Sep-Pak Vac C18, 10 g, Waters). The retained material was eluted with a mixture of methanol and 0.1% TFA in water. A linear gradient from 0 to 100% methanol in 55 min, at 5 ml/min, was used. Fractions were taken every 5 min, and analyzed by UPLC. Fractions containing the desired compounds were evaporated *in vacuo*, and dissolved in a small volume of a mixture of DMSO and methanol (50:50). Products were purified by preparative HPLC using a SunFire C18 column (10 μm, 10 × 150 mm, Waters). Compounds were chromatographed with mixtures of acetonitrile and 0.1% TFA in water, in isocratic conditions optimized for each compound, at 5 ml/min.

### Structural Characterization of Compounds

LC/MS (liquid chromatography mass spectrometry) analyses were carried out on an Agilent 1200 Rapid Resolution HPLC system equipped with a SB-C8 column (2.1 × 30 mm, Zorbax) and coupled to a Bruker maXis mass spectrometer. Samples were subjected to LC/ESI-TOF analysis in order to determine their molecular formula. For the Nuclear Magnetic Resonance (NMR) analysis samples were dissolved in deuterated methanol (CD_3_OD) and transferred to a 1.7 mm tube. Acquisitions were carried out on a Bruker AVANCE III 500 MHz spectrometer equipped with a 1.7 mm TCI Microcryoprobe. All spectra were recorded at 297 K. Structural elucidation of compounds was carried out by analysis of a combination of 1D (^1^H and ^13^C), and 2D (^1^H-^1^H COSY, TOCSY, HSQC and HMBC) NMR experiments.

### Determination of Stereochemistry of Argimycins PI and PII

A solution containing argimycins PI/PII was subjected to a desulfurization/reduction reaction using the method reported by Martin et al. (2004) with some modifications: a solution of argimycins PI and PII (1.0 mg) and nickel chloride (2.0 mg) in 2.0 mL of MeOH/H_2_O was added to a screwcap flask containing NaBH_4_ (2.0 mg) with immediate resealing of the reaction vial. A black precipitate, namely Ni_2_B, was immediately formed and the mixture stirred for 1h at 50°C. After centrifugation, the supernatant containing the released *N*-acetylalanine was recovered, evaporated to dryness and subjected to the Marfey’s analysis (Marfey, 1984): 0.4 mg of the reaction product were suspended in 1.0 mL of 12 N HCl and heated at 110°C for 2 h. The crude hydrolysate was evaporated to dryness under a nitrogen stream, and the residue was dissolved in 100 μL of miliQ H_2_O. A 1% (w/v) solution (100 μL) of L-FDVA [*N*-(2,4-dinitro-5-fluorophenyl)-valinamide] in acetone was added. After addition of 20 μL of 1 M NaHCO_3_ solution, the mixture was incubated at 40°C for 60 min. The reaction was quenched by addition of 10 μL of 1 N HCl, and the crude mixture was diluted with 700 μL of acetonitrile and analyzed by LC/MS on an Agilent 1100 single quadrupole. Similarly, the standards of L- and D-alanine were also separately derivatized L-FDVA, according to the method mentioned above. Separations were carried out on an Agilent Zorbax SB-C8 column (2.1 × 30 mm, 3.5 μm) maintained at 40°C. A mixture of two solvents, A (10% acetronitrile, 90% water) and B (90% acetonitrile, 10% water), both containing 1.3 mM trifluoroacetic acid and 1.3 mM ammonium formiate, was used as the mobile phase under a linear gradient elution mode (10-40% B in 11 min, then 100% B) at a flow rate of 0.3 mL/min.

## Results

### Genome Mining of *Streptomyces argillaceus* ATCC 12956 Genome

*Streptomyces argillaceus* ATCC 12956 genomic DNA was subjected to 454 sequencing, yielding 512,452 paired end sequences with a mean of 340.62 nt (174.55 Mb total). *De novo* assembly of these sequences resulted in 1538 contigs, 1330 of which were larger than 500 nucleotides. The N50 of the contig assembly was around 10.5 Kb, being the largest around 68.1 Kb. Most of these contigs were ordered in 20 scaffolds: the N50 of the scaffolding was 1.2 Mb and the largest scaffold was 3.5 Mb. This combination of scaffolds and contigs resulted in an estimated genome size of 10.7 Mb. Genome analysis led to the annotation of 7638 coding sequences, 4 rRNAs and 66 tRNAs. Sequence analysis with antiSMASH (Blin et al., 2013; Medema et al., 2015; Weber et al., 2015) predicted the existence of 31 biosynthetic gene clusters (BGCs), including five for PKs, two for non-ribosomal peptides (NRPs), three for hybrid PK-NRPs, seven for ribosomally synthesized and post-translationally modified peptides (RiPPs) and four for terpenes (**Table 2**).

**Table 2:** Secondary metabolite gene clusters identified in *S. argillaceus* by genome mining.

Cluster	Type	Predicted product
1	Butyrolactone	Unknown
2	Thiopeptide	Lactazole-like
3	Type III PKS	Unknown
4	Other	Unknown
5	Other	Unknown
6	Oligosaccharide-Type II PKS	Mithramycin
7	Bacteriocin	Unknown
8	Type II PKS	Putative spore pigment
9	NRPS	Unknown
10	Ectoine	Hidroxyectoine
11	TransAT-PKS-NRPS	Unknown
12	Melanin	Melanin
13	Siderophore	Desferrioxamine
14	NRPS	Unknown
15	Terpene	Albaflavenone
16	Lantipeptide	Unknown
17	Siderophore	Unknown
18	Type I PKS	Argimycins P^(a)^
19	Bacteriocin	Unknown
20	Butylolactone-terpene	Gamma-butyrolactone/geosmine
21	Siderophore	Unknown
22	Terpene	Hopene
23	Type I PKS	Unknown
24	Bacteriocin	Unknown
25	Melanin	Melanin
26	Terpene	Isorenieratene
27	PKS-NRPS	Antimycins
28	Lantipeptide-NRPS	Unknown
29	Lantipeptide	Unknown
30	Other	Unknown
31	PKS-NRPS	Unknown

*^(a)^this work; PKS, polyketide synthase; NRPS, non-ribosomal peptide synthetase.*

Among PK clusters, there are two for type II PKS: cluster 6 corresponds to the already characterized mithramycin gene cluster (Lombó et al., 2006); and cluster 8 most probably is involved in the biosynthesis of a spore pigment. At the time of analysis, no prediction was available for compounds encoded by type I (clusters 18 and 23) and type III (cluster 3) PK clusters. The PK-NRP cluster 27 was predicted to be involved in antimycin biosynthesis, since it shows strong similarity to antimycin clusters that are highly conserved in other microorganisms (Seipke et al., 2011), while no assumption could be done for the PK-NRP clusters 11 and 31. Based on the strong similarities to other terpene biosynthesis gene clusters, cluster 15 was predicted to code for albaflavenone, described in *S. coelicolor* (Zhao et al., 2008); cluster 20 contains homologous genes to *sabRAS*, involved in gamma-butyrolactone biosynthesis in *S. acidiscabies* (Healy et al., 2009), and a geosmine synthase homologous gene that has been reported to be the only gene required for geosmine biosynthesis (Cane et al., 2006); cluster 22 was similar to hopene biosynthesis gene clusters (Ghimire et al., 2015); and cluster 26 would code the isorenieratene carotenoid (Takano et al., 2005). Some other clusters showed similarity to already known clusters, which has allowed predicting the corresponding biosynthesis products. For example, cluster 2 might be involved in the biosynthesis of a lactazole thiopeptide, similar to that described in *S. lactacystinaeus* (Hayashi et al., 2014). Also, cluster 10 would code for hydroxyectoine, described in other organisms such as *S. chrysomallus* (Prabhu et al., 2004); and cluster 13 that contains all genes required for the biosynthesis of siderophore desferrioxamine, described in *S. coelicolor* (Barona-Gómez et al., 2004). On the other hand, some of the clusters showed high similarity to very well conserved clusters, the metabolic products of which are not known. This is the case of cluster 9 (NRPs), and clusters 17 and 21 (NRPS-independent siderophores).

### Analysis of Cluster 18 and Identification of Encoded Compounds

**FIGURE 1:**
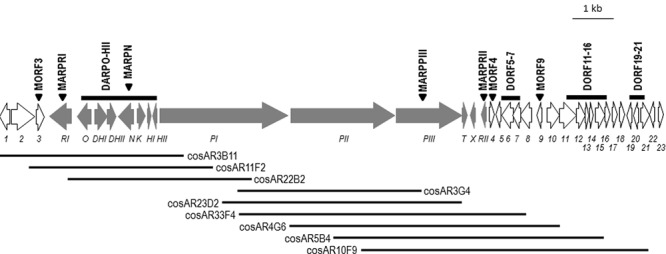
**Genetic organization of cluster 18.** Genes are shown to scale. Argimycins P biosynthesis genes (*arp*) are shown in gray. Other genes are shown in white and are numbered. Triangles indicate those genes that have been inactivated. Bars indicated DNA regions that have been deleted. Black lines represent overlapping cosmids isolated from this chromosomal DNA region.

Among the 31 *S. argillaceus* identified clusters, cluster 18 was selected for further characterization. This cluster spanned 68.295 kb and contained 37 open reading frames (*orf*), including three coding for a type I PKS, seven for regulatory genes and several for tailoring enzymes (**Figure 1**; **Table 3**). According to **Table 3**, ArpPI to ArpPIII PKS show strongest homology to the three PKS from the streptazone E biosynthesis gene cluster (Ohno et al., 2015). Co-linearity of cluster 18 was confirmed using PCR probes from different DNA regions of the cluster, to identify overlapping cosmids from an *S. argillaceus* gene library, followed by sequencing the ends of DNA inserts in these cosmids (**Figure 1**). Sequence of this cluster has been deposited at the European Nucleotide Archive (EBI-ENA) under the accession number LT615255, and at MIBIG under the accession number BGC0001433.

**Table 3:** Functions of gene products from cluster 18.

Gene	aa	Proposed function	Similar protein (acc. number)	Identical aa (%)
Orf1	360	SARP-like transcriptional regulator	WP_037724676.1	80
Orf2	773	Xre-like transcriptional regulator	WP_061914503.1	80
Orf3	254	Hypothetical protein	WP_031129425.1	81
ArpRI	828	SARP-like transcriptional regulator	WP_051790319.1	81
ArpO	483	Dehydrogenase	WP_030346790.1	87
ArpDHI	392	Dehydrogenase/hydroxylase	WP_030346791.1	96
ArpDHII	293	Imine reductase	WP_030346792.1	96
ArpN	534	Aminotransferase	WP_030346793.1	90
ArpK	182	Flavin reductase	WP_037814843.1	93
ArpHI	137	Cyclase	WP_030346795.1	95
ArpHII	136	Cyclase	WP_030346796.1	92
ArpPI	4625	Type I Polyketide Synthase	BAT51065.1	75
ArpPII	3575	Type I Polyketide Synthase	BAT51066.1	69
ArpPIII	2203	Type I Polyketide Synthase	BAT51067.1	73
ArpT	257	Type II thioesterase	WP_030348965.1	86
ArpX	177	Unknown	WP_031487542.1	88
ArpRII	210	TetR-like transcriptional repressor	WP_019065342.1	79
Orf4	215	Reductase	WP_030993716.1	84
Orf5	126	Hypothetical protein	WP_030579546.1	63
Orf6	381	Oxidase	WP_024490800.1	75
Orf7	212	Putative transcriptional repressor	WP_063624787.1	63
Orf8	381	Monooxygenase	WP_051386635.1	63
Orf9	167	*N*-acetyltransferase	WP_024490799.1	49
Orf10	406	Cytochrome P450	WP_024490798.1	82
Orf11	559	AMP-dependent synthetase and ligase	WP_037777637.1	80
Orf12	285	Hypothetical protein	WP_024490796.1	90
Orf13	170	Thioesterase	WP_024490795.1	84
Orf14	96	Acyl carrier protein	WP_052488578.1	65
Orf15	307	Methyltransferase	WP_037777640.1	82
Orf16	291	Thioesterase	WP_024490792.1	87
Orf17	234	Phosphopantetheinyl transferase	WP_024490791.1	54
Orf18	208	Pyrophosphorylase	WP_028437185.1	48
Orf19	137	Dehydratase	WP_051387014.1	74
Orf20	134	Thioesterase	WP_051387012.1	80
Orf21	333	3-oxoacyl-ACP synthase	WP_040020314.1	75
Orf22	161	Xre-like transcriptional regulator	WP_024495143.1	77
Orf23	163	LuxR-like transcriptional regulator	WP_061444915.1	63

To identify the biosynthesis product(s) of this cryptic gene cluster, two approaches were followed in parallel: (i) Generation of a mutant in a PKS gene. To completely block the biosynthesis pathway directed by cluster 18, the PKS gene *arpPIII* was disrupted by inserting pBSKTT1701 into this gene, generating mutant MARPPIII (**Table 1**; Supplementary Figure S1); (ii) Inactivation of the putative *tetR*-like transcriptional repressor gene *arpRII*. Cluster 18 contains seven putative transcriptional regulatory genes: two coding for SARP-like activators (*orf1* and *arpRI*), two for Xre-like regulators (*orf2* and *orf22*), one for a FMN binding repressor (*orf7*), and one for a *tetR*-like repressor (*arpRII*). This last one (*arpRII*) was located two *orfs* downstream of PKS genes. Since TetR regulators usually behave as transcriptional repressors (Ramos et al., 2005), *arpRII* was inactivated to increase production of compounds encoded by the cluster, which will facilitate their identification. Using pHZMutTetR, most of *arpRII* was replaced by an apramycin resistance cassette generating mutant MARPRII (**Table 1**; Supplementary Figure S2).

**FIGURE 2:**
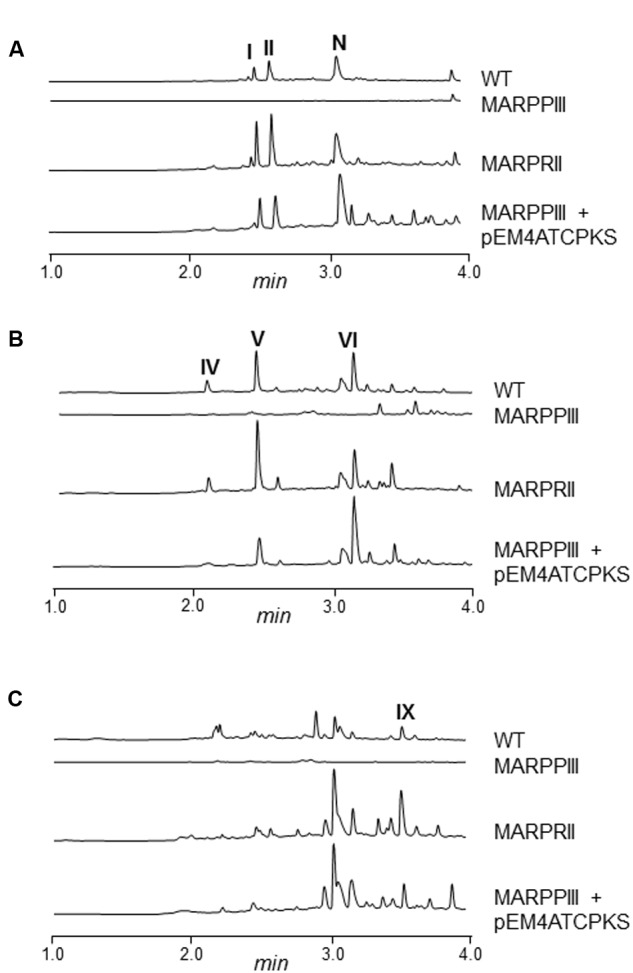
**UPLC chromatograms of *S. argillaceus* wild type and mutant strains in *arp* genes.** Butanol extracts of *S. argillaceus* wild type strain (WT), *S. argillaceus* MARPPIII, *S. argillaceus* MARPRII, and *S. argillaceus* MARPPIII (pEM4ATCPKS). Chromatograms are shown at 400 nm **(A)**, 272 nm **(B)** and 230 nm **(C)**. Peaks corresponding to the different argimycins P are indicated as follows: argimycins PI and PII (**I** and **II**); nigrifactin (**N**); argimycin PIV (**IV**); argimycin PV (**V**); argimycin PVI (**VI**); and argimycin PIX (**IX**).

Mutants MARPPIII and MARPRII (and the wild type strain as control) were cultivated in R5A medium to compare the metabolite profiles of these strains. Culture samples were harvested along growth and extracted with different solvents (ethyl acetate; ethyl acetate and 0.1% formic acid; *n*-butanol; or chloroform). Extracts were run by UPLC and chromatograms were obtained at 400, 272, and 230 nm, and analyzed by identifying peaks that disappeared in MARPPIII and increased in MARPRII, in relation to the wild type strain. Chromatograms of *n*-butanol extracts showed differential peaks between strains (data not shown). Since the corresponding compounds were mostly produced in very low amounts, a media screening was conducted to increase their production levels. SM10 medium was selected as the best medium and was used thereafter. **Figure 2** shows UPLC analysis of *n*-butanol extracts of MARPPIII and MARPRII cultivated in SM10, in comparison to those from the wild type strain. As it can be observed, inactivation of *arpPIII* (*S. argillaceus* MARPPIII) led to the disappearance of peaks present in the wild type strain: at 400 nm (**Figure 2A**, peaks **I, II** and **N**), 272 nm (**Figure 2B**, peaks **IV, V** and **VI**) and 230 nm (**Figure 2C**, peak **IX**). HPLC-MS analysis of the corresponding compounds revealed *m/z* values in positive mode of 331 for peaks **I** and **II**; 176 for peaks **N** and **VI**; 208 for peak **IV**; 192 for peak **V**; and 178 for peak **IX**. Detection of these compounds was recovered by expressing *arpPIII* (plus *arpT*) under the control of the erythromycin resistance promoter (pEM4ATCPKS), into MARPPIII (**Figures 2A–C**). On the other hand, inactivation of putative repressor gene *arpRII* (mutant MARPRII) led to a clear increase of production of most of these compounds (**Figures 2A–C**). From all these results it was deduced that compounds from those peaks were encoded by cluster 18. In addition, analysis of the three subunits of type I PKS encoded by cluster 18 suggested that the PK chain synthesized by this enzyme would result from the condensation of six malonyl-CoA units. Consequently, strains with higher intracellular concentration of malonyl-CoA should produce higher amounts of these compounds. Therefore, *S. argillaceus* GIH (overexpressing the acetyl-CoA carboxylase *ovmGIH* genes), *S. argillaceus* AFTA (a mutant in the acyl-CoA:diacylglycerol acyltransferase *aftAa* gene) and *S. argillaceus* AFTA-GIH (*S. argillaceus* AFTA overexpressing the acetyl-CoA carboxylase genes), which accumulate higher concentrations of malonyl-CoA (Zabala et al., 2013), were tested for production of compounds corresponding to peaks **I** and **II** (331 mass). As expected, higher production of these compounds was obtained with all strains, being the highest increases with *S. argillaceus* AFTA-GIH (**Table 4**).

**Table 4:** Productions of peaks I and II in *S. argillaceus* strains containing higher intracellular concentration of malonyl-CoA.

Strain	Peak I^∗^	Peak II^∗^
WT	43188 ± 1871	55614 ± 2979
GIH	55395 ± 11477	63914 ± 9257
AFTA	85358 ± 4543	102456 ± 5311
AFTA-GIH	151616 ± 18711	171609 ± 7102

*^∗^Area of peaks, in arbitrary units.*

### Purification, Structural Elucidation and Bioactivity of Compounds Encoded by Cluster 18

**FIGURE 3:**
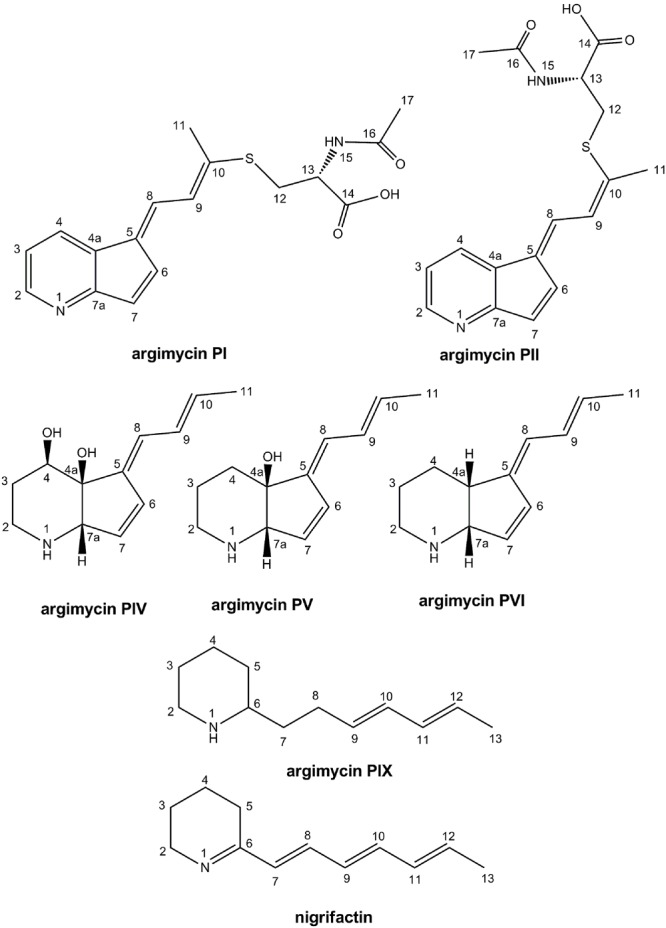
**Chemical structures of argimycins P (relative configuration shown for argimycins PI, PII, PIV, PV, and PVI)**.

Compounds from those peaks mentioned above were purified by preparative HPLC. Although peaks **I** and **II** were purified independently, they both correspond to a mixture of two main compounds that were present in different proportions. The major compound in peak **I** was named argimycin PI, and that in peak **II** argimycin PII. Moreover, there was a gradual interconversion of argimycin PII into argimycin PI and vice versa along time, till reach an equilibrium in which both compounds were present in the same proportion in both samples. Yields obtained for the different compounds were as follows: 3.5 mg (peak **I**); 3.2 mg (peak **II**); 3.9 mg (peak **N**, nigrifactin/named argimycin PIII); 8.8 mg (peak **IV**, named argimycin PIV); 1.9 mg (peak **V**, named argimycin PV); 0.9 mg (peak **VI**, named argimycin PVI); and 1 mg (peak **IX**, named argimycin PIX). The structure of these compounds, subsequently named argimycins PI-IX, were elucidated by NMR and MS analyses (**Figure 3**).

Argimycin PI shows a molecular formula C_17_H_18_N_2_O_3_S on the basis of the molecular ion *m/z* 331.1109 obtained by ESI-TOF ([M+ H]^+^, calcd. for C_17_H_19_N_2_O_3_S^+^, 331.1116), that additionally displayed the typical isotope pattern for compounds that contain a sulfur atom. Sulfur has an isotopic distribution showing three isotopes, ^32^S, ^33^S and ^34^S, with a ratio 100/0.8/4.5. Therefore, compounds containing a sulfur atom show in their MS spectra an M+2 ion with an increased intensity with respect to non-sulfur containing molecules. Supplementary Figure S3 shows the experimental and simulated (for a molecular formula of C_17_H_19_N_2_O_3_S) HRMS spectra of argimycin PI, indicating agreement with the presence of a sulfur atom in the molecule (not the case if the simulated formula was C_17_H_19_N_2_O_4_). The same molecular formula was determined for argimycin PII, based on the observed molecular ion *m/z* 331.1112 and the identical isotope pattern as argimycin PI. MS/MS experiments in ESI+ mode revealed, for both molecules, that the parent ion (C_17_H_18_N_2_S + H^+^) generated a C_12_H_12_NS^+^ fragment, and in turn, loss of SH_2_ provided the C_12_H_10_N^+^ ion. Fragmentation under ESI- conditions provided ion [M-H]^-^ (*m/z* 329) as base peak, suggesting the presence of an acidic proton. NMR analysis (**Table 5**) showed 7 aromatic/olefinic protons, one methylene and one methine, both of them bound to heteroatom and two single methyl groups. Based on this data, together with the coupling constants from ^1^H-NMR and information from bidimensional COSY, HSQC, ^13^C-HMBC ^15^N-HMBC and NOESY, connectivity and stereochemistry of argimycin PI and PII was determined (Supplementary Figures S4–S11). Supplementary Figure S12 shows the signal assignments and the correlations observed by NOESY. For argimycin PII the NOESY experiment, which showed interaction between H8 and H4, while H9 interacts with H6 and Me-11, proved key to determine the correct stereochemistry of the double bonds (Supplementary Figure S13). The connectivity of the two isomeric argimycins PI and PII explains the results of the MS experiments. Under ESI- mode, both molecules ionize as [M-H]- (*m/z* 329), in agreement with the carboxylic acid function. Also, under ESI-, a fragment C_12_H_10_NS^-^ (*m/z* 200) is observed, which can be explained by a decarboxylation shown in Supplementary Figure S14. The stereochemistry of the *N*-acetylcysteine contained in these compounds was unambiguously established as ‘L’ by means of a previous desulfurization/reduction reaction to release *N*-acetylalanine, and further hydrolysis and analysis of the released amino acid derivatized with L-FDVA. Under the reported conditions, the retention times (min) for the derivatized alanine standards were 4.79 (L-Ala) and 6.87 (D-Ala), being the retention time for the observed peak in the HPLC trace of the L-FDVA derivatized hydrolysis product of argimycins PI/PII 4.78 min, coincident with the retention time for L-Ala-L-FDVA (see Supplementary Figure S15).

**Table 5:** NMR data for argimycins PI-PII (^1^H, 500 MHz; ^13^C, 150 MHz, in CD_3_OD)

	Argimycin PI		Argimycin PII
Carbon	δ_H_ (mult, *J*, Hz)	δ_C_ (ppm)	δ_H_ (mult, *J*, Hz)
2	8.30 (d, 5.2)	143.6	8.30 (d, 5.2)
3	7.27 (dd, 7.7, 5.2)	119.2	7.24 (dd, 7.7, 5.2)
4	8.25 (d, 7.7)	127.9	8.12 (d, 7.7)
4a	–	131.9	–
5	–	n.d.	–
6	7.67 (d, 5.6)	130.9	7.43 (d, 5.6)
7	7.00 (d, 5.6)	127.4	6.98 (d, 5.6)
7a	–	159.1	–
8	7.69 (d, 11.8)	128.5	7.80 (d, 11.8)
9	6.96 (d, 11.8)	117.3	7.03 (d, 11.8)
10	–	148.2	–
11	2.33 (br s) 3H	17.6	2.38 (br s) 3H
12	3.62 (dd, 13.0, 4.6) 3.27 (dd, 13.0, 7.7)	32.4	3.49 (dd, 13.0, 4.6) 3.23 (dd, 13.0, 7.7)
13	4.74 (dd, 7.7, 4.7)	52.1	4.61 (dd, 7.7, 4.7)
14	–	172.3	–
16	–	171.7	–
17	2.01 (s) 3H	21.0	1.99 (s) 3H

**Nitrogen**	**δ_N_ (ppm)**		

1	259.1		
15	121.9		

**Table 6** shows the NMR signal assignments for argimycin PIV, argimycin PV and argimycin PVI. Argimycin PV possesses a molecular formula of C_12_H_17_NO, according to the base peak *m/z* 192.1383 by ESI-TOF ([M+ H]^+^, calcd. for C_12_H_18_NO^+^, 192.1388). The ^1^H-NMR spectrum showed five olefinic protons and a methyl group signal, which was split into a doublet (Supplementary Figure S16). Comparative analyses using databases [Dictionary of Natural Products (DNP) NMR features] with the molecular formula and the structural characteristics mentioned, failed to dereplicate any possible candidate, hinting to the novelty of its structure. Together with bidimensional NMR and ^13^C-NMR spectra, the assignment provided in **Table 6** was established (positions numerated as in argimycins PI and PII). Double bond relative stereochemistry for the ring fusion was established as *cis* (between the OH in 4a and the H in 7a) by comparison with the chemical shifts and NOESY correlation profile observed for argimycin PVI, whose stereochemistry was unambiguously determined (see below).

**Table 6:** NMR data for argimycins PIV-PVI (^1^H, 500 MHz; ^13^C, 150 MHz, in CD_3_OD).

	Argimycin PIV		Argimycin PV		Argimycin PVI	
Carbon	δ_H_ (mult, *J*, Hz)	δ_C_ (ppm)	δ_H_ (mult, *J*, Hz)	δ_C_ (ppm)	δ_H_ (mult, *J*, Hz)	δ_C_ (ppm)
2	3.27 (app quintet, ca. 6.3)3.02 (app quintet, ca. 6.3)	35.5	3.18 (app quintet, ca. 6.3)3.07 (app quintet, ca. 6.3)	39.1	3.13 (m)3.00 (m)	40.3
3	1.97 (m) 1.72 (m)	23.3	1.95 (m) 1.68 (m)	16.4	1.78 (m) 1.69 (m)	17.6
4	3.89 (dd, 6.6, 3.0)	68.9	1.89 (m)	30.9	1.99 (m)	21.3
4a	–	77.4	–	76.3	3.02 (m)	39.1
5	–	142.4	–	144.8	–	140.5
6	7.06 (br d, 6.0)	135.5	7.02 (br d, 6.0)	134.5	7.17 (br d, 5.8)	137.2
7	6.02 (br d, 6.0)	126.6	6.00 (br d, 6.0)	126.7	6.15 (br dt, 5.9)	129.3
7a	4.36 (br s)	67.0	4.13 (br s)	66.9	4.25 (dd, 6.5, 2.3)	59.4
8	6.15 (d, 11.2)	125.2	6.10 (d, 11.2)	123.0	6.04 (d, 11.1)	123.4
9	6.49 (ddq, 14, 11.2, 1.5)	127.5	6.47 (ddq, 14.0, 11.2, 1.6)	127.4	6.52 (ddq, 14, 11.1, 1.6)	127.6
10	5.91 (dq, 14, 6.7)	132.1	5.88 (dq, 14.0, 6.8)	131.5	5.83 (dq, 14, 6.8)	130.2
11	1.85 (br d, 6.7)	17.1	1.84 (dd, 6.9, 1)	17.1	1.83 (brd, 6.9)	16.9

Argimycin PVI possesses a molecular formula of C_12_H_17_N, according to the base peak *m/z* 176.1433 by ESI-TOF ([M+ H]^+^, calcd. for C_12_H_18_N^+^, 176.1439). UV/vis spectrum shows a similar profile, including the maximum at 272 nm, as in argimycin PV. Comparison of the ^1^H-NMR spectra for argimycin PV and argimycin PVI (Supplementary Figure S16) indicates that both molecules are structurally related, the difference in molecular formula suggests that argimycin PVI is the dehydroxy analog of argimycin PV. Dereplication based on molecular formula and NMR signals (DNP NMR features) failed to identify any previously described product, confirming the novelty of the structure. Bidimensional NMR spectra (Supplementary Figure S17) were acquired to elucidate the structure, assigning the signals unambiguously (**Table 6**). Double bond stereochemistry was established based on the coupling constants (identical profile as argimycin PV). NOESY spectrum showed a correlation between H-4a and H-7a, confirming the fused ring *cis* stereochemistry.

Argimycin PIV possesses a molecular formula of C_12_H_17_NO_2_, according to the base peak *m/z* 208.1334 by ESI-TOF ([M+ H]^+^, calcd. for C_12_H_18_NO_2_^+^, 208.1338). UV/vis spectrum shows a similar profile, including the maximum at 272 nm, as in argimycin PV and argimycin PIV. Comparison of the ^1^H-NMR spectra for argimycin PVI in CD_3_OD and DMSO-*d*_6_ showed that the former provides better signal resolution (Supplementary Figure S18), and CD_3_OD was selected for elucidation. This also facilitated comparison with argimycin PV and argimycin PVI (Supplementary Figure S19), which indicates that all three molecules are structurally related, the difference in molecular formula suggests that argimycin PIV is a hydroxylated analog of argimycin PV. As before, dereplication based on molecular formula and NMR signals (DNP NMR features) failed to identify any previously described product, confirming the novelty of the structure. Bidimensional NMR spectra (Supplementary Figure S20) were acquired to elucidate the structure, assigning the signals unambiguously (**Table 6**). Double bond stereochemistry was established based on the coupling constants (identical profile as argimycin PV). NOESY spectrum showed a correlation between H-4a and H-7a, confirming the fused ring *cis* stereochemistry. Relative stereochemistry in C4 was established by the analysis of the coupling constant *J* at H4 (dd, 6.6Hz, 3.0Hz). 3D modeling (hydroxyls in relative *cis* position) shows dihedral angles (H4-H3a) = -170° and (H4-H3b) = -55°, compatible with the observed *J* values. On the other hand, the modeling of the epimer at H4 shows dihedral angles (H4-H3a) = -59° and (H4-H3b) = +59°, incompatible with the observed *J* values (Supplementary Figure S21).

Argimycin PIII (nigrifactin) possesses a molecular formula of C_12_H_17_N, according to the base peak *m/z* 176.1438 by ESI-TOF ([M+H]^+^, calcd. for C_12_H_18_N^+^, 176.1439). UV/vis spectrum shows a different profile, including a maximum at 350 nm. Comparison of the ^1^H-NMR spectra for argimycin PIII in CD_3_OD and DMSO-*d*_6_ showed that the later provides better signal resolution (Supplementary Figure S22), but CD_3_OD was selected for elucidation in order to facilitate comparison with argimycin PIV, argimycin PV and argimycin PVI (Supplementary Figure S23). This comparison clearly shows that argimycin PIII is not structurally similar to the other three fused bicyclic argimycins P. In this case, dereplication based on molecular formula and NMR signals (DNP NMR features) provided a candidate, the previously described alkaloid nigrifactin (UV/vis max at 350 nm), which indeed is biosynthetically related to the argimycins PIV-PVI. Further analysis including bidimensional NMR spectra permitted the elucidation and signal assignment described in **Table 7**. All-*trans* stereochemistry of the double bonds was confirmed by analysis of coupling constants.

**Table 7:** NMR data for argimycin PIII (nigrifactin) and argimycin PIX (^1^H, 500 MHz; ^13^C, 150 MHz, in CD_3_OD).

	Argimycin PIII (nigrifactin)		Argimycin PIX	
Carbon	δ_H_ (mult, *J*, Hz)	δ_C_ (ppm)	δ_H_ (mult, *J*, Hz)	δ_C_ (ppm)
2	3.70 (m)	44.0	3.36 (br dt, 13.0) 3.00 (td, 13.0, 2.6)	44.4
3	1.94 (m)	19.5	1.85 (m) 1.65 (m)	22.1
4	1.91 (m)	16.4	1.90 (m) 1.56 (qt, 12.9, 2.8)	21.6
5	3.02 (m)	24.4	2.05 (br dt, 14.0) 1.40 (br dd, 13.0 2.9)	28.0
6	–	178.5	3.06 (m)	56.0
7	6.43 (d, 15)	121.3	1.72 (m) 1.66 (m)	33.1
8	7.58 (dd, 15, 11)	149.5	2.20 (m)	27.3
9	6.45 (dd, 13, 11)	127.7	5.52 (ddd, 14.0, 7.0)	128.4
10	6.91 (dd, 14,11)	146.6	6.08 (dd, ca 14.0, 12.0)	131.7
11	6.34 (dd, 14, 11)	131.3	6.04 (dd, ca 14.0, 12.0)	131.1
12	6.20 (m)	139.0	5.64 (dq, 14.0, 6.8)	127.3
13	1.90 (br d, 7.0)	17.5	1.73 (br, d, 6.8)	16.7

Argimycin PIX possesses a molecular formula of C_12_H_21_N, according to the base peak *m/z* [M+H]^+^ observed at 180.1753 (calcd. for C_12_H_22_N^+^ = 180.1747). Searching this molecular formula in the DNP retrieved just one hit, 2-octylpyrrole which was not compatible with the NMR spectrum observed for argimycin PIX. The molecular formula contains two degrees of unsaturation less than nigrifactin (C_12_H_17_N), which agrees with a structure similar to that of nigrifactin with two double bonds less, reducing the conjugation level in agreement with the observed UV maximum at 225 nm (compared to the 352 nm for nigrifactin). The ^1^H-NMR (Supplementary Figure S24) and HSQC (Supplementary Figure S25) spectra showed four olefinic methine groups, one aliphatic N-bound methine, one methyl doublet very similar to that of nigrifactin and six aliphatic methylene groups (one of them bound to N). All these features were in agreement with the preliminary hypothesis of a partially reduced nigrifactin structure. Further analysis of the whole set of 2D NMR spectra (including COSY and HMBC) allowed the elucidation of the compound structure. The configuration of double bonds was established by analysis of the coupling constants, permitting the elucidation and signal assignment described in **Table 7**.

**FIGURE 4:**
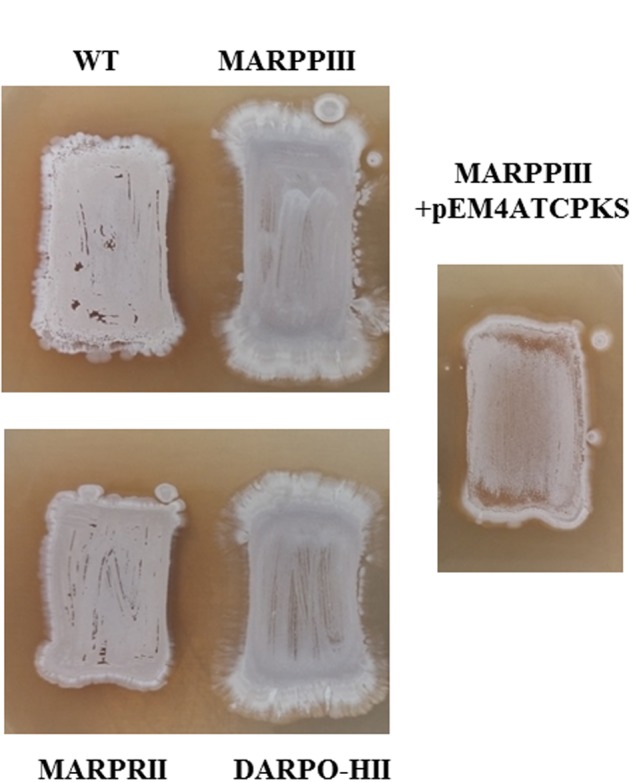
**Morphology of *S. argillaceus* strains: argimycins P producers (wild type, WT; MARPRII); non-producer mutants (MARPPIII; DARPO-HII); and complemented mutant (MARPPIII + pEM4TCPKS)**.

Cytotoxic activity of argimycins P was evaluated against a panel of 59 tumor cell lines, using 10 μM concentrations. None of them showed cytotoxic activity (data not shown). In antibiotic activity tests nigrifactin (argimycin PIII), argimycins PI and PII, and argimycin PVI showed weak antibiotic activity against *Micrococcus luteus* (data not shown). On the other hand, production of argimycins P exerts some effect on *S. argillaceus* development. Growth on agar plates of argimycins P producer and non-producer strains revealed that non-producer mutants *S. argillaceus* MARPPIII and *S. argillaceus* DARPO-HII grow and sporulate better than producer strains (wild type and the overproducer strain *S. argillaceus* MARPRII). Moreover, after complementing S. *argillaceus* MARPPIII (*S. argillaceus* MARPPIII-pEM4ATCPKS) this phenotype was reversed (**Figure 4**). This suggests that argimycins P play some role in colony development in *S. argillaceus.*

### Limits of *arp* Biosynthesis Gene Cluster

Since cluster 18 was shown to encode argimycins P, this cluster was renamed as cluster *arp*. Limits of the *arp* cluster were established by comparing genes (and their gene products) located at each end of the cluster with others in data-bases, together with inactivating single genes and/or deleting set of genes located at both ends (**Table 1, Figure 1**; Supplementary Figures S26–S32), followed by analysis of argimycins P production in mutant strains (Supplementary Figure S33).

At the left end of the cluster, a gene coding for an unknown protein (*orf3*) and several others coding for regulatory proteins (*orf1, orf2* and *arpRI*) were located. *orf1* to *orf3* were homologous to genes in other *Streptomyces* that were not involved in the biosynthesis of secondary metabolites, either showing the same (*Streptomyces* sp. NRRL WC-3719) or slightly different genetic organization (as in *Streptomyces* sp. 769, *Streptomyces* sp. MspMP-M5 and *S. natalensis* ATCC 27448). This suggested that these genes were not involved in argimycins P biosynthesis. To confirm this, *orf3* was inactivated by gene replacement. Using pHZMutorf3, the wild type copy of *orf3* was replaced by an apramycin resistance cassette, generating mutant MORF3. Analysis of cultures of mutant MORF3 revealed that still produces argimycins P, confirming that *orf3* was not necessary for argimycin P biosynthesis. To establish the left boundary of the cluster, inactivation of *arpRI* was carried out. Using pHZMutSARP mutant MARPRI was generated by gene replacement. No argimycins P were detected in cultures of MARPRI, proving the involvement of *arpRI* in argimycins P biosynthesis. Therefore, the left boundary of *arp* cluster was established as *arpRI* (**Figure 1**).

On the right end of the cluster there were genes coding for enzymes and regulatory proteins that could be involved in argimycins P biosynthesis. To establish the right end of the *arp* cluster, deletion and inactivation of specific genes were carried out in a sequential way, starting from the far 3′-end of the cluster. First, *orf19* to *orf21* were deleted and replaced by an apramycin resistance cassette using pHZdel2. Since the resultant mutant DORF19-21 still produced argimycins P, *orf11* to *orf16* were deleted next. This DNA region included *orf11*, which codes for a putative AMP-dependent syntethase and ligase that could be involved in activating the cysteine residue that is present in argimycins PI and PII. Using pHZDel13 mutant DORF11-16 was generated and still produced argimycins P, including argimycins PI and PII, indicating that this region is not required for argimycins P biosynthesis and specifically, that *orf11* is not involved in activating the cysteine residue for the biosynthesis of argimycin PI and PII. *orf9* codes for an *N*-acetyl-transferase that was envisioned to be involved in transferring the acetyl group to the cysteine residue in argimycins PI and PII. Therefore, *orf9* was inactivated next. Using plasmid pHZMutNAcTr, mutant MORF9 was generated and still produced argimycins P, including PI and PII. This indicated that the *orf9* gene product was not the acetyl-transferase involved in the biosynthesis of these argimycins P. Deletion of *orf5* to *orf7* (includes a transcriptional repressor and an oxidase) and inactivation of *orf4* (coding for a reductase) were carried out next. Using plasmids pHZDel1820 and pHZMutorf17, mutants DORF5-7 and MORF4 were generated by gene replacement. UPLC analysis of culture extracts from these mutants showed that in both cases argimycins P were still produced. All these results and the mentioned above with MARPRII mutant, indicated that *orf4* to *orf23* were not necessary for argimycins P biosynthesis, and established the right end of the cluster at *arpRII* (**Figure 1**). In addition, these results showed that neither *orf9* nor *orf11* were required for the formation of NAC moiety of argimycins PI and PII.

### Analysis of Argimycin P Biosynthesis Gene Cluster and Proposed Biosynthesis Pathway

The *arp* cluster spanned 45.282 kb and includes fourteen genes: two coding for regulatory proteins (*arpRI* and *arpRII*), eleven for structural proteins (*arpPI, arpPII, arpPIII, arpT, arpN, arpDHI, arpDHII, arpHI, arpHII, arpO*, and *arpK*), and one for a hypothetical protein (*arpX*) (**Figure 1**; **Table 3**).

**FIGURE 5:**
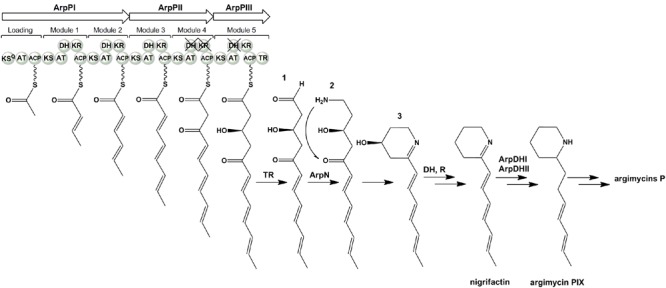
**Proposed biosynthesis pathway for argimycins P**.

The **ArpP** type I modular PKS would synthesize a 12-carbon PK backbone (**Figure 5**). It is constituted by three subunits: ArpPI containing the loading domain (LD) and extension modules 1 (M1) and 2 (M2); ArpPII with the third and fourth extension modules (M3 and M4); and ArpPIII with the fifth extension module (M5) and a thioester reductase domain (TR) (**Figure 5**). All five extension modules and the LD contain a β-ketoacyl synthase (KS), an acyl carrier protein (ACP) and acyltransferase (AT) domain. KS domains will be responsible for the decarboxylative Claisen condensations of six acyl-CoA units to form the PK chain. The KS domains from M1 to M5 contain the catalytic triad CHH (Xu et al., 2013). In the LD, Gln is replacing Cys within the conserved amino acid region AQSSS, indicating that the starter unit in the polyketide biosynthesis would be malonyl-CoA (Bisang et al., 1999). All ArpP ACPs contain the conserved Ser residue to which the phosphopantetheine arm is linked (Donadio and Katz, 1992). The ArpP AT domains contain the conserved amino acid region GHSxG around the catalytic Ser residue, and are predicted to accept malonyl-CoA as substrate since they carry signature motifs associated with malonyl-CoA substrate specificity (Haydock et al., 1995; Reeves et al., 2001; Yadav et al., 2003): presence of a branched hydrophobic residue (Ile) beyond the catalytic Ser; and a HAFH motif that includes the catalytic His. All five extender modules in ArpP PKS contain a KR and a DH domain. All ArpP KR domains contain the NADP(H)-binding motif and the catalytic triad KSY (Reid et al., 2003). KRs are usually of B1 type when they work together with processing enzymes like DHs (Keatinge-Clay, 2007). Based on sequence fingerprints in the LDD loop, catalytic region and Lid (Caffrey, 2003; Keatinge-Clay, 2007), all ArpP KRs except KR4, could be classified into the B1 type. Interestingly, in KR4 the conserved LDD motif is not preserved: there is an Asn residue instead of the strictly conserved Asp. Its absence could indicate that this KR is of A-type. However, A-type KRs usually possess a Trp residue N-terminal to the catalytic Tyr that is absent in KR4 (Caffrey, 2003; Keatinge-Clay, 2007). This fact could indicate a malfunctioning of KR4 that could lead to a keto group at C8 in the nascent polyketide chain (**Figure 5**). All ArpP modules contain a DH domain. DH1, DH2 and DH3 show the four hallmark DH motifs, including those around the catalytic residues His (HxxxGxxxxP) and Asp (DxxxQ/H) (Keatinge-Clay, 2008). However, in DH4 and DH5 two of these motifs are lacking, which indicates that DH4 and DH5 are inactive. Products of B-type KRs are dehydrated to *trans*-double-bonds by the corresponding DH (Xu et al., 2013). Accordingly, the polyene side chain of nigrifactin (argimycin PIII), a putative early shunt product in argimycins P biosynthesis, which would result from the activity of ArpP and ArpN (see below), shows three trans-double-bonds. Typically, the release of the full length polyketide chain is catalyzed by a thioesterase domain (TE) located at the C-terminal of the last module. However, in the ArpP PKS there is a TR domain instead that would be responsible for the NAD(P)H-dependent reductive release of the acyl thioester attached to the adjacent ACP domain (Du and Lou, 2010) to yield the putative aldehyde product **1** (**Figure 5**). This TR domain shows the consensus motifs usually found in this type of domains, both in NRPS and PKS (Konz and Marahiel, 1999).

**ArpT** shows similarity to type II thioesterases such as TamB (ADC79640.1; 47% identical amino acids) and TrdB (ADY38534.1; 47% identical amino acids from tirandamycin gene clusters in *Streptomyces* sp. 307-9 (Carlson et al., 2010) and *Streptomyces* sp. SCSIO1666 (Mo et al., 2011), respectively; and ScoT (NP_630685.1; 53% identical amino acids) from the coelimycin P1 gene cluster in *S. coelicolor* M145 (Pawlik et al., 2007; Gómez-Escribano et al., 2012). These proteins are editing proteins that eliminate aberrant polyketide growing chains from PKS, thus allowing its normal correct function (Heathcote et al., 2001; Mo et al., 2011).

**FIGURE 6:**
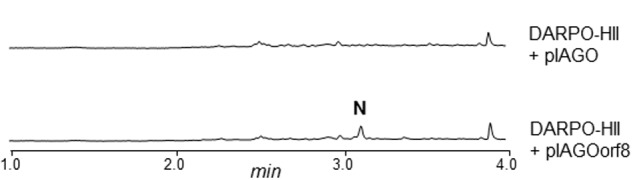
**UPLC chromatograms at 350 nm of butanol extracts of *S. argillaceus* DARPO-HII and *S. argillaceus* DARPO-HII (pIAGOorf8)**. N, nigrifactin.

**ArpN** shows similarity to class III aminotransferases such as CpkG (67% identical amino acids) that is involved in the biosynthesis of coelimycin P1 in *S. coelicolor* (Pawlik et al., 2007; Gómez-Escribano et al., 2012). These pyridoxal phosphate (PLP)-dependent enzymes can catalyze the transfer of an amino group from an amino donor to an aldehyde group. Accordingly, ArpN is proposed to catalyze the amination of compound **1** (**Figure 5**). The resultant compound **2** could spontaneously cyclize or could suffer other modifications before ring formation. For example, in the biosynthesis of coelimycin P1 it has been proposed that the transamination step is followed by several epoxidations and oxidation before cyclization of the PK chain. To understand the formation of the piperidine ring during the biosynthesis of argimycins P, *arpN* was inactivated using pHZMutAT (**Table 1**; Supplementary Figure S34). The resultant mutant *S. argillaceus* MARPN neither produced any argimycin P nor accumulated a new compound, which indicated that ArpN was involved in the biosynthesis of argimycins P and suggested its participation at an early step (Supplementary Figure S35). Then, a second mutant was generated by deleting *arpO* to *arpHII* genes, using pHZDel59b (**Table 1**; Supplementary Figure S36). As expected, the resultant mutant *S. argillaceus* DARPO-HII didn’t produce any argimycin P (**Figure 6**). When *arpN* was expressed in this mutant, the resultant strain *S. argillaceus* DARPO-HII pIAGOorf8 produced nigrifactin (**Figure 6**). These results indicate that biosynthesis of nigrifactin only requires the action of ArpP PKS and aminotransferase ArpN, and suggests that compound **2** suffers a spontaneous cyclization to generate compound **3**. Dehydration and reduction of this compound would result in the formation of nigrifactin (**Figure 5**). Moreover, these results also indicate that the other structural genes in the cluster (*arpDHI, arpDHII, arpHI, arpHII, arpK*, and *arpO*) must be involved in later steps in the biosynthesis pathway leading to the formation of argimycins PI/PII, PIV, PV, PVI and PIX.

**ArpDHI** and **ArpDHII** show high similarity to several dehydrogenases. ArpDHI was similar to hypothetical acyl-CoA dehydrogenases. It contains the Acyl-CoA dehydrogenase, C-terminal domain (pfam080028). These proteins catalyze FAD-dependent dehydrogenation steps. It also contains the conserved protein domain NcnH (cd01159). This is a hydroxylase involved in the biosynthesis of polyketide naphthocyclinone that hydroxylates several substrates such as aloesaponarin II and SEK26 (Brünker et al., 2001). We propose ArpDHI could hydroxylate the piperidine ring and/or reduce the C7-C8 double bond of the polyene chain during the biosynthesis of argimycins P. The identification of argimycin PIX that lacks the C7-C8 double bond in the polyene chain (**Figure 3**) supports the existence of a dehydrogenase acting at the polyene chain.

**ArpDHII** is similar to putative 6-phosphogluconate dehydrogenases. It belongs to the Rossmann fold superfamily, and contains the NAD_binding_2 domain of 6-phosphogluconate dehydrogenases (pfam03446) and the NAD(P) binding domain of Shikimate dehydrogenase (cd01065). In addition, a Blastp search with ArpDHII using the Protein Data Bank proteins (PDB) gave significant alignments with several imine reductases, such as Q1EAE0 (acc. number 3ZGY_A) from *Streptomyces kanamyceticus* (Rodríguez-Mata et al., 2013) and AoIRED (acc. number 5A9S_B) from *Amycolatopsis orientalis* (Aleku et al., 2016). We suggest that ArpDHII could be an imine reductase that catalyzes the reduction of the imine group of argimycins P.

**ArpHI** and **ArpHII** belong to the NTF2_like superfamily, and contain a SnoaL-like domain (pfam12680 and pfam13474, respectively). SnoaL is a polyketide cyclase which catalyzes the last cyclization step during nogalamycin biosynthesis (Sultana et al., 2004). ArpHI shows similarity to hypothetical proteins, while ArpHII was similar to several putative hydrolases. In addition, ArpHI and ArpHII were similar to StzF and StzE, respectively. These two proteins have recently been proposed to act as cyclases during streptazone E biosynthesis (Ohno et al., 2015). We propose ArpHI and ArpHII to be involved in the formation of the five-membered ring present in some argimycins P.

**ArpO** shows high similarity to several putative oxidases. It contains a NAD(P)-binding Rossmann domain (pfam13450) and a putative FAD-binding dehydrogenase domain (PRK12834). Also, it contains domains that belong to the conserved protein domain families desat_CrtD (TIGR02733) and CrtI_fam (TIGR02734). These enzymes catalyze desaturation/dehydrogenation reactions. According to that, ArpO could carry out dehydrogenation/oxidation reactions during biosynthesis of argimycins P.

**ArpK** shows high similarity to flavin reductases, and contains flavin reductase like domains (smart00903 and pfam01613). ArpK could be involved in regenerating flavin nucleotides that could be used by putative dehydrogenases ArpDHI and ArpO.

The *arp* gene cluster contains two regulatory genes located at the boundaries of the cluster. ***arpRI*** codes for a SARP-like regulatory protein (Wietzorrek and Bibb, 1997). At its N-terminus contains a Trans_reg_C (Pfam00486), followed by a BTAD (*Bacterial Transcriptional Activation Domain)* (cd158319) domain; and at its C-terminus, the ATP-binding domain AAA_16 (Pfam13191). ArpRI behaves as a transcriptional activator. Its inactivation completely blocked argimycins P production, as it was shown in *S. argillaceus* MARPRI (see above). ***arpRII*** codes for a transcriptional regulator belonging to the TetR family (Ramos et al., 2005). It contains the TetR domain (pfam00440). As shown above, ArpRII behaves as a transcriptional repressor, since its inactivation led to an increase in argimycins P production (*S. argillaceus* MARPRII). In addition, the *arp* cluster contains a gene (***arpX***), whose product shows high similarity to hypothetical proteins from different *Streptomyces*. At this moment, no role can be assigned to this gene product in argimycins P biosynthesis.

**FIGURE 7:**
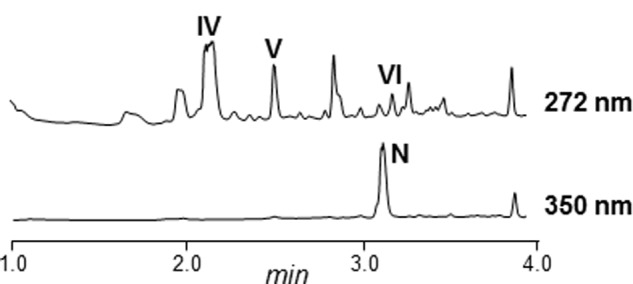
**UPLC chromatograms of butanol extracts of *Streptomyces* NRRL S-1022.** N, nigrifactin; IV, argimycin PIV; V, argimycin PV; VI, argimycin PVI.

Most of the *arp* gene products show high similarity to proteins from an incomplete and uncharacterized PKS gene cluster in *Streptomyces* sp. NRRL S-1022 (**Table 3**; Supplementary Figure S37). Specifically, there was high similarity and synteny between upstream genes of the PKS region, and also with the *arpT* gene product. Upstream of *arpRI* and downstream of *arpT*, the similarity is lost, that is in *Streptomyces* sp. NRRL S-1022 there are no homologous genes to *arpX* and *arpRII*. To determine if this strain also produces argimycins P, the strain was obtained from the NRRL culture collection and cultivated. Butanol extracts of cultures of this strain showed production of argimycin PIII (nigrifactin), argimycin PIV, argimycin PV and argimycin PVI (**Figure 7**). This confirmed that genes *arpRI* to *arpT* are sufficient to synthesize these argimycins P. However, no argimycin PI and PII was detected (data not shown), reinforcing the hypothesis that enzymes required for the formation and incorporation of NAC moiety to these compounds are encoded by genes outside the *arp* cluster.

## Discussion

The first genome sequences obtained from *Streptomyces* revealed that these microorganisms had greater potential to produce secondary metabolites than expected (Bentley et al., 2002; Ikeda et al., 2003). The improvement and development of sequencing technologies and bioinformatic tools has allowed the use of genome mining as a new and effective approach to discover new metabolites produced by microorganism, by activating and/or increasing production of unknown compounds encoded by cryptic biosynthesis gene clusters (Olano et al., 2014; Rutledge and Challis, 2015; Ochi, 2016). Genome mining applied to the antitumor mithramycin producer *S. argillaceus* has allowed the identification of 31 gene clusters for the biosynthesis of secondary metabolites. In addition to the mithramycin gene cluster, which has been previously characterized (Lombó et al., 2006), the metabolic products of 11 gene clusters could be predicted. Cluster 18 (renamed as *arp* cluster) was related to the coelimycin P1 and the recently reported streptazone E gene cluster (Gómez-Escribano et al., 2012; Ohno et al., 2015), and was predicted to direct the biosynthesis of a PK derived from the condensation of six malonyl-CoA units. Identification of compounds encoded by this cluster was carried out by combining several strategies: (i) inactivation of the type I PKS gene *arpPIII* to block production of compounds; (ii) inactivation of the putative TetR-transcriptional repressor *arpRII* to increase production of compounds; (iii) cultivation of wild type and mutant strains in different production media, to increase production and identification of compounds; and (iv) use of strains with increased intracellular amounts of malonyl-CoA, to favor the biosynthesis of the malonyl-CoA derived PK chain. Using this combined strategy seven compounds were identified, one of them, nigrifactin, previously identified in *Streptomyces* FFD-101(Terashima et al., 1969). The remaining newly discovered compounds were named argimycins P. Nigrifactin and argimycin PIX are simple piperidine alkaloids consisting of a piperidine ring with a polyene chain attached. They differ in the nitrogen atom that is reduced in argimycin IX, and in the existence of a C7-C8 double bond in the polyene chain in nigrifactin absent in argimycin PIX. Argimycin PIV, argimycin PV and argimycin PVI differ in their hydroxylation pattern, and contain a piperidine ring fused to a five-membered ring and a shorter polyene side chain. They are structurally related to pyrindicin (Onda et al., 1973) and streptazones E and F (Liu et al., 2013), and to other alkaloids with shorter side chains such as abikoviromycin (latumcidin) (Umezawa et al., 1951; Sakagami et al., 1958). Argimycins PI and PII are isomers, and unlike argimycins PIV-PVI contain a pyridine ring, and additionally, they contain a NAC residue attached to the polyene chain. For some of the piperidine alkaloid compounds structurally related to argimycins P antimicrobial, cytotoxic and antiviral activities has been reported (Umezawa et al., 1951; Puder et al., 2001). In some of these compounds, bioactivity has been related to the presence of double bonds, an epoxy group, or an imine group in the piperidine ring, among others (Hegde et al., 1994; Puder et al., 2000; Maruyama et al., 2003; Liu et al., 2013). In addition, some of these and related compounds have been shown to exert other activities such as decreasing blood pressure, inhibiting biosynthesis of cholesterol or having analgesic properties (Terashima et al., 1970; Grabley et al., 1991). Some argimycins P also showed weak antibiotic but no cytotoxic activity, being argimycin PIII (nigrifactin) the most active one. If they display other activities is unknown at this time and remains to be discovered. On the other hand, argimycins P seem to play a role in growth and colony development in *S. argillaceus*: argimycins P non-producer mutants show better growth and sporulation than producer strains. Therefore, expression of the *arp* cluster and/or production of the encoded argimycins P seem to reduce/decrease colony growth and development in *S. argillaceus*. Further studies will be required to understand the role of these genes and compounds on these processes.

The boundaries of the *arp* biosynthesis gene cluster have been determined by inactivating/deleting genes at both ends of the cluster. It contains 14 genes, including two coding for regulatory proteins, 11 for structural proteins and one for a hypothetical protein. The *arp* cluster shows high similarity to a partial and uncharacterized cluster from *Streptomyces* NRRL S-1022, and to the recently published *stz* gene cluster from *Streptomyces* sp. MSC090213JE08 (Ohno et al., 2015). The genes *arpX* and *arpRII* are absent in those strains. The hypothetical role of *arpX* is unclear. In the case of *arpRII*, we have shown that codes for a putative TetR-like repressor of argimycins P biosynthesis, since its inactivation greatly increases argimycins P production. In the case of *Streptomyces* sp. MSC090213JE08, there is no evidence of argimycin P production, although nigrifactin has been proposed as a biosynthesis intermediate (Ohno et al., 2015). However, in this manuscript we have shown that *Streptomyces* NRRL S-1022 actually produces most argimycins P, which indicates that those homologous genes between *S. argillaceus* and S-1022 strains are sufficient for the biosynthesis of the piperidine ring containing argimycin P compounds. The pyridine ring containing compounds argimycin PI and argimycin PII haven’t been identified in cultures of S-1022 strain, which might indicate that enzymes for generating these compounds are encoded by genes located outside of the *arp* cluster, and are strain specific.

Based on the structure of argimycins P and on the bioinformatic analysis of the *arp* cluster, a pathway for the early steps in argimycins P biosynthesis is proposed (**Figure 5**). This would start by the condensation of six malonyl-CoA units by PKS ArpP. According to the presence of KR and DH domains in modules 1 to 3 of ArpP, the first three elongation steps would be followed by the ketoreduction and dehydration of the resulting β-keto groups to render *trans*-double bonds at the corresponding sites. Considering that the KR domain of module 4 as well as the DH domains of modules 4 and 5 are inactive, the resulting PK chain would contain a β-keto group at C8 and a hydroxyl group at C10. Since module 5 contains a TR domain, this PK chain would be released to render the putative aldehyde **1**. This compound would be the substrate for aminotransferase ArpN, rendering the hypothetical product **2** that can spontaneously cyclize to form the piperidine ring. In this report, we have shown that formation of this ring in *S. argillaceus* only requires ArpP and ArpN. We propose that the reaction product of these enzymes would be compound **3** (**Figure 5**). Piperidine compounds with a hydroxyl group at C4 but with a shorter side chain have been identified in cultures of piperidine-producing organisms (Grabley et al., 1991; Groenhagen et al., 2014). Spontaneous dehydration and reduction of compound **3** would render nigrifactin (**Figure 5**). Its reduction at the C7-C8 double bond and at the imine group by ArpDHI and ArpDHII, would result in the formation of argimycin PIX. Late biosynthetic steps would involve formation of the fused five-membered ring and incorporation of NAC adduct, among others. Candidates for the cyclization reaction could be ArpHI/ArpHII, which contain a domain present in the SnoaL cyclase from the nogalamycin pathway (Sultana et al., 2004), and are similar to two putative cyclases from the streptazone E pathway (Ohno et al., 2015). Within the *arp* cluster there are no candidate genes for formation and transfer of NAC. Downstream of the cluster, there are two genes (*orf11* and *orf9*) that were initially proposed to be involved in this process. However, as mentioned above, inactivation of these genes did not abolish argimycins PI/PII production. In coelimycin P1 biosynthesis pathway, Gómez-Escribano et al. (2012) have hypothesized that incorporation of the NAC adduct would take place by ring opening of bis-epoxide of a biosynthesis intermediate *via* nucleophilic attack of the NAC thiol group. In the case of argimycins P biosynthesis pathway, it is possible that ArpO, which is similar to different oxidases, could be involved in oxidizing the side chain, being the resultant product substrate for the incorporation of NAC. Future studies would be required to clarify biosynthetic steps downstream of the formation of piperidine ring.

## Conclusion

By genome mining of *S. argillaceus* we have identified the type I cryptic *arp* gene cluster and the encoded argimycins P family of new compounds, which in addition to antibiotic activity, they seem to play a role in colony growth and development of this strain.

### Notes in Proof

Very recently, it has been shown that the TR domains of the PKS and the ω-aminotransferases (which are similar to ArpP-TR and ArpN respectively) involved in the biosynthesis of polyketide alkaloids coelimycin P1 and cyclizidine, are responsible for the release of the polyketide chain as an aldehyde and its subsequent transamination (Awodi et al., 2016; Peng et al., 2016). On the other hand, Peng et al. (2016) have shown that 3-hydroxyl-6-methyl-piperidine (an analog of compound **3**) is the substrate for imine reduction, and it is unstable being readily transformed in a dehydrated product.

## Author Contributions

CM, JS, and FM conceived and designed the project; SY, DZ, and JC conducted experiments; AB carried out compound purifications; SY, BM, CO, and CM performed sequence *in silico* analysis; CM wrote the manuscript, and FM and JS contributed to preparing the final version of the paper. All authors read and approved the final manuscript.

## Conflict of Interest Statement

The authors declare that the research was conducted in the absence of any commercial or financial relationships that could be construed as a potential conflict of interest.

The reviewer MN and handling Editor declared their shared affiliation, and the handling Editor states that the process nevertheless met the standards of a fair and objective review.
