# Supplementary material for: Identification by Genome Mining of a Type I Polyketide Gene Cluster from Streptomyces argillaceus Involved in the Biosynthesis of Pyridine and Piperidine Alkaloids Argimycins P
Source: Front Microbiol. 2017 Feb 10;8:194. doi: 10.3389/fmicb.2017.00194 (PMC5300972; doi:10.3389/fmicb.2017.00194)

Supplementary Material

Identification by genome mining of a type I polyketide gene cluster from *Streptomyces argillaceus* involved in the biosynthesis of pyridine and piperidine alkaloids argimycins P

Suhui Ye[1], Brian Molloy[1], Alfredo F. Braña[1], Daniel Zabala[1], Carlos Olano[1], Jesús Cortés[2], Francisco Morís[2], José A. Salas[1] and Carmen Méndez[1]*

*** Correspondence:** Carmen Méndez: cmendezf@uniovi.es

COMPOSITION OF PRODUCTION MEDIA

SM10: MOPS, 20.9 g/l; L-proline, 11.5 g/l; glycerol, 23 g/l; NaCl, 0.5 g/l; K_2HPO_4, 2.1 g/l; Na_2SO_4, 0.28 g/l; $MgSO_4$ 0.02 M, 10 ml/l; $CaCl_2$ 0.02M, 10 ml/l; and 5 ml/l of an oligoelement solution ($ZnCl_2$, 0.04 g/l; $FeCl_3{\cdot}6H_2O$, 0.2 g/l; $CuCl_2{\cdot}2H_2O$, 0.1 g/l; $MnCl_2{\cdot}4H_2O$, 0.01 g/l; $Na_2B_4O_7{\cdot}10H_2O$, 0.01 g/l; $(NH_4)_6Mo_7O_{24}{\cdot}4H_2O$, 0.01 g/l); pH 6.5.

SM17: glucose, 2 g/l; glycerol, 40 g/l; starch, 2 g/l; soy protein, 5 g/l; bactopeptone 5 g/l; yeast extract, 5 g/l; NaCl 5 g/l; $CaCO_3$, 2 g/l; tap water; pH 6.4

PLASMID CONSTRUCTS TO GENERATE MUTANTS:

Several plasmids were constructed using oligoprimers from Table S1, to generate mutants by gene disruption or gene replacement:

pHZMutorf3: a 2.0 kb DNA fragment containing the 3'-end of *orf2* and the 5'-end of *orf3* was amplified using oligonucleotides Mutorf3_1_A and Mutorf3_1_B, digested with EcoRI, and subcloned in the right orientation into the same site of pUO9090, generating pUOMutorf3_1. Also, a 2.0 kb DNA fragment containing the 3'-end of *orf3* and the 3'-end of *arpRI* was amplified using oligonucleotides Mutorf3_2_A and Mutorf3_2_B, digested with EcoRV and XbaI and subcloned into the same sites of pUOMutorf3_1, downstream of the apramycin resistance cassette, generating pUOMutorf3. Finally, the insert was rescued from this construct as a SpeI fragment and subcloned into the XbaI site of pHZ1358.

pHZMutSARP: a 1887 bp DNA fragment containing the 3'-end of *arpRI* and *orf3* was amplified using oligonucleotides MutSARP_2_A and MutSARP_2_B, digested with BglII and XbaI, and subcloned into the BamHI and XbaI sites of pUO9090, downstream of the apramycin resistance cassette, generating pUOMutSARP_2. Also, a 1992 bp DNA fragment containing the 5'-end of *arpRI* and the 3'-end of *arpO* was amplified using oligonucleotides MutSARP_1_A and MutSARP_1_B, digested with BglII and HindIII and subcloned into the same sites of pUOMutSARP_2, upstream of the apramycin resistance cassette, generating pUOMutSARP. Finally, the insert was rescued as an SpeI fragment and subcloned into the XbaI site of pHZ1358.

pHZDel59b: a 1781 bp DNA fragment containing the 5'-ends of *arpHII* and *arpPI*, was amplified using oligonucleotides Mutorf9b_1_A and Mutorf9b_1_B, digested with HindIII and PstI, and subcloned into the same sites of pUO9090, upstream of the apramycin resistance cassette, generating pUOMutorf9b_1. Also, a 1991 bp DNA fragment containing *arpRI* and the 3'-end of *arpO*, was amplified using oligonucleotides Mutorf5_2_A and Mutorf5_2_B, digested with BglII and XbaI, and subcloned into the BamHI and XbaI sites of pUOMutorf9b_1, downstream of the apramycin resistance cassette, generating pUODel59b. Finally, the insert from this construct was rescued as an SpeI fragment and subcloned into the XbaI site of pHZ1358.

pHZMutAT: a 2078 bp DNA fragment containing *arpK*, *arpHI*, *arpHII* and the 5'-end of *arpN*, was amplified using oligonucleotides MutAT1_A and MutAT1_B, digested with BamHI and HindIII, and subcloned into the BglII and HindIII sites of pUO9090, upstream of the apramycin resistance

cassette, generating pUOMutAT1. Also, a 1464 bp DNA fragment containing *arpDHII* and the 3'-end of *arpN*, was amplified using oligonucleotides MutAT2_A_bis and MutAT2_B_bis, digested with EcoRV and XbaI, and subcloned into the same sites of pUOMutAT1, downstream of the apramycin resistance cassette, generating pUOMutAT. Finally, the insert was rescued as an SpeI fragment and subcloned into the XbaI site of pHZ1358.

<u>pBSKTT1701</u>: a 996 bp DNA fragment from *arpPIII* was amplified using oligonucleotides SA1701A and SA1701B, subcloned into pCRBlunt, rescued as an EcoRI- HindIII fragment and subcloned into the same sites of pBSKTT.

<u>pHZMutTetR</u>: a 1795 bp DNA fragment containing *arpX* and the 3'-ends of *arpT* and *arpRII*, was amplified using oligonucleotides MutTetR2_A and MutTetR2_B, digested with BamHI and EcoRV, and subcloned into the same sites of pUO9090, downstream of the apramycin resistance cassette, generating pUOMutTetR2. Also, a 1830 bp DNA fragment containing *orf4*, *orf5*, the 5'-end of *arpRII* and the 3'-end of *orf6*, was amplified using oligonucleotides MutTetR1_A and MutTetR1_B, subcloned into pCRBlunt, rescued as a 1650 bp EcoRI fragment, and subcloned in the right orientation, into the EcoRI site of pUOMutTetR2, generating pUOMutTetR. Finally, the insert was rescued from this construct as an SpeI fragment and subcloned into the XbaI site of pHZ1358.

<u>pHZMutorf17</u>: a 1854 bp DNA fragment containing *arpRII* and the 5'-ends of *arpX* and *orf4*, was amplified using oligonucleotides Mutorf17_1_A and Mutorf17_1_B, digested with BglII, and subcloned in the right orientation in the same site of pUO9090, upstream of the apramycin resistance cassette, generating pUOMtorf17_1. Also, a 1972 bp DNA fragment containing *orf5*, *orf6* and the 3'-end of *orf4*, was amplified using oligonucleotides Mutorf17_2_A and MutNReg2_A, subcloned into pCRBlunt, rescued as an EcoRV fragment, and subcloned in the right orientation in the same site of pUOMtorf17_1, downstream of the apramycin resistance cassette, generating pUOMutorf17. Finally, the insert from this construct was rescued as an SpeI fragment and subcloned into the XbaI site of pHZ1358.

<u>pHZDel1820</u>: a 1988 bp DNA fragment containing *orf8* and the 5'-end of *orf7*, was amplified using oligonucleotides MutNReg1_A and MutNReg1_B, digested with BglII and HindIII, and subcloned into the same sites of pUO9090, upstream of the apramycin resistance cassette, generating pUODel1820_1. Also, a 1505 bp DNA fragment containing *orf4*, the 3'-end of *orf5* and the 5'-end of *arpRII*, was amplified using oligonucleotides Del1820_2_A and MutTetR1_B, subcloned into pCRBlunt, rescued as an EcoRV fragment and subcloned in the right orientation, into the same site of pUODel1820_1, downstream of the apramycin resistance cassette, generating pUODel1820. Finally, the whole insert was rescued from this construct as an SpeI fragment and subcloned into the XbaI site of pHZ1358.

<u>pHZDel2</u>: a 2004 bp DNA fragment containing *orf17* and *orf18*, and the 3'-ends of *orf19* and *orf16*, was amplified using oligonucleotides Del2_1A and Del2_1B, digested with BamHI and HindIII, and subcloned into the BglII-HindIII sites of pUO9090, upstream of the apramycin resistance cassette, generating pUODel2_1. Also, a 2017 bp DNA fragment containing *orf22, orf23* and the 3'-end of *orf21*, was amplified using oligonucleotides Del2_2A and Del2_2B, digested with BamHI and XbaI and subcloned into the same sites of pUODel2_1, generating pUODel_2. Finally, the insert from this construct was rescued as an SpeI fragment and subcloned into the XbaI site of pHZ1358.

pHZMutNAcTr: a 1950 bp DNA fragment containing *orf10* and the 5'-ends of *orf9* and *orf11* was amplified using oligonucleotides MutNAcTr1_A and MutNAcTr1_B, digested with EcoRI and HindIII and subcloned into the same sites of pUO9090, upstream of the apramycin resistance cassette, generating pUOMutNAcTr1. Also, a 1983 bp DNA fragment containing *orf8* and the 3'-end of *orf9*, was amplified using oligonucleotides MutNAcTr2_A and MutNAcTr2_B, digested with BamHI and EcoRV and subcloned into the same sites of pUOMutNAcTr1, downstream of the apramycin resistance cassette, generating pUOMutNAcTr. Finally, the insert in pUOMutNAcTr was rescued as an SpeI fragment and subcloned into the XbaI site of pHZ1358.

pHZDel3: a 1950 bp DNA fragment containing *orf10* and the 5'-ends of *orf11* and *orf9*, was amplified using oligonucleotides MutNAcTr_1_A and MutNAcTr_1_B, digested with EcoRI and subcloned in the right orientation, into the same site of pUO9090, upstream of the apramycin resistance cassette, generating pUODel3_1. Also, a 2004 bp DNA fragment containing *orf17* and *orf18*, and the 3'-ends of *orf19* and *orf16*, was amplified using oligonucleotides Del2_1A and Del2_1B, subcloned in pCRBlunt, rescued as a BamHI-EcoRV DNA fragment and subcloned into the same sites of pUODel3_1, downstream of the apramycin resistance cassette, generating pUODel3. Finally, the whole insert in this construct was rescued as an SpeI fragment and subcloned into the XbaI site of pHZ1358.

PLASMID CONSTRUCTS FOR GENE EXPRESSION

Several plasmids were constructed using oligoprimers from Table S1, to express *arp* genes:

pIAGOorf8bis: a 1753 bp DNA fragment containing *arpN* was amplified using oligonucleotides Orf8_Abis and Orf8_B, digested with SpeI and XbaI, and subcloned in the right orientation into the XbaI site of pIAGO.

pEM4ATCPKS: a 8073 bp DNA fragment containing *arpPIII* and *arpT* was amplified using oligonucleotides PKScompMut_A and PKScompMut_B, digested with EcoRV, and subcloned in the right orientation into the end-filled BamHI site of pEM4ATC.

Table S1: oligonucleotides used for PCR

PRIMER	SEQUENCE 5’-3’
**PRIMERS DESIGNED TO GENERATE MUTANTS**	
**Del2_1A**	AAAGGATCCGACACCATGAAGGGCTACTG
**Del2_1B**	GGGAAGCTTAAGATCGGTGGTGAACTCG
**Del2_2A**	AAAGGATCCTGGTCAAGAACATGAAGCT
**Del2_2B**	AAATCTAGATGGGGCACTGAAGTATGTG
**MutNAcTr1_B**	ATAAAGCTTCATGCGTTCCAGTGCTGT
**MutNAcTr1_A**	AATGAATTCGGGTTCTGCTCCATGAGG
**MutNReg1_A**	AAAAGATCTGCCGTAGAACAGTTCGTGGT
**MutNReg1_B**	AAAAAGCTTAGGTCGGTATCGGGCGGTAT
**Del1820_2_A**	AAAGATATCAACGTCCCGAACCTGACCGA
**MutTetR1_B**	AAGCTTAAGGTGGAGTAGAGGCTGGA
**Mutorf9b_1_A**	AAAAAGCTTGCCGGTGACGATCCAC
**Mutorf9b_1_B**	AGGCTGCAGTGAAGGCGGCGACATAAC
**Mutorf5_2_A**	AAAAGATCTCAAGGTGCTCTCCGTCAAG
**Mutorf5_2_B**	AAATCTAGAGCAGTGAGAAGACGACCGTA
**MutNAcTr1_A**	AATGAATTCGGGTTCTGCTCCATGAGG
**MutNAcTr1_B**	ATAAAGCTTCATGCGTTCCAGTGCTGT
**MutNAcTr2_A**	AAAGGATCCCGACACCGTCAGGATCA
**MutNAcTr2_B**	AAAGATATCTGGACAGCAGGGTCGAGG
**Mutorf17_1_A**	TTTAGATCTCGGGGTGCTGACTGATCT
**Mutorf17_1_B**	AAAAGATCTATGATCGCGTCCGCTTC
**Mutorf17_2_A**	TTTGATATCTTCGACCTCCAGCGCGTCAA
**MutReg2_A**	AAAGGATCCACAGAGCGGTCCAGCACTT
**Mutorf3_1_A**	AAAGAATTCAGATCGCGCAGACCTACC
**Mutorf3_1_B**	TTTGAATTCCGCCGAGTTCGACGTAGT
**Mutorf3_2_A**	AATAGATCTCCGCCTTCGACTTCTTCC
**Mutorf3_2_B**	AAATCTAGAGAAGAACGGCACGACGAC

**MutSARP_1_A**	AAAAGATCTAGCACGTTCTCGGGACTG
**MutSARP_1_B**	AAAAAGCTTGTTCTGCAGGGTGGTCAACT
**MutSARP_2_A**	AAAAGATCTGTATCCCCCTCACCGTCAT
**MutSARP_2_B**	AAATCTAGAGGCCTGCTGATCATGGATAC
**SA1701A**	GCTGGATCCGTCGGCCTTCGTGCTGTTCT
**SA1701B**	GGTAAGCTTCGTCCCAGACGCGGTAGTA
**MutTetR_1_A**	AGATCTGGCAGGACCTGTCACTCTTC
**MutTetR_1_B**	AAGCTTAAGGTGGAGTAGAGGCTGGA
**MutTetR_2_A**	GGATCCTACGGGGTCAACCTGCGCTC
**MutTetR_2_B**	GATATCCTGGACGACCCCGAACTGCT
**MutAT1_A**	ATTGGATCCTTCGGCGTAGTTGTCACC
**MutAT1_B**	AAAAAGCTTGGGTGCAGGGAGAACT
**MutAT2_A_bis**	AAAGATATCCTGATGCTCGGTCTGGAGTT
**MutAT2_B_bis**	AAATCTAGATGAGGAGCAAGTCAATGTCG
**PRIMERS DESIGNED TO EXPRESS GENES**	
**PKScompMut_A**	AAAGATATCACTGAGGAGAGACGAACC
**PKScompMut_B**	AAAGATATCTGAAAAGGCGTCACGACT
**Orf8_A bis**	GGGACTAGTCGCATTCGGTCATGTT
**Orf8_B**	AAATCTAGAGGTCGTCCCGTCCGTTAC
**PRIMERS DESIGNED TO VERIFIED MUTANTS**	
**Del2_A_comp**	AGCGAACCGTTCCTGAAA
**ApraC_rv**	TCATTCTGTGGGCCGTAC
**Del2_B_comp**	GTCCCCACAATAAATGCCC
**ApraC_fw**	TCATTCTGTGGGCCGTAC
**Del3_A_comp**	ATTCGCCCAACACGGTGT
**ApraC_rv**	TCATTCTGTGGGCCGTAC
**Del3_B_comp**	GAACGCGCCATCACGAA
**ApraC_fw**	TCATTCTGTGGGCCGTAC

**MutNATr2_A**	AAAGGATCCCGACACCGTCAGGATCA
**ApraC_rv**	TCATTCTGTGGGCCGTAC
**1701orf4RT_A**	GAGTTGCCCCTGCTCCTC
**ApraC_fw**	TCATTCTGTGGGCCGTAC
**MutNAcTrC_A**	AGGCCGTTCGGTTTCAG
**MutNAcTrC_B**	CTTGCGGAGGAAAATTGC
**Del1820_2_A**	AAAGATATCAACGTCCCGAACCTGACCGA
**MutTetR_1_B**	AAGCTTAAGGTGGAGTAGAGGCTGGA
**Orf3_A**	AACGGATCCATGTCATCGCCCTTCCCT
**Orf3_B**	AAGGAATTCGGCGGGGAGTTTGGGACT
**378orf1Abis**	GCAGAATTCCTGTGGAGCGTCGAAGAA
**378orf1B**	ATTGAATTCTTCCGAGTGGCGTGCC
**SA1701FW**	GTCTCGACGCCGTGC
**M13 FW**	GTAAAACGACGGCCAG
**MutTetRcompr_A**	AGACCTCGCCCGCTATCT
**MutTetRcompr_B**	GCCGTAAGCAGGAAGAACC
**Mutorf6_1_A**	AAATCTAGAGTTCTCCCGGGGAAGTGA
**MutAT1_A**	ATTGGATTCTTCGGCGTAGTTGTCACC
**Orf8_A bis**	GGGACTAGTCGCATTCGGTCATGTT
**Orf8_B**	AAATCTAGAGGTCGTCCCGTCCGTTAC
**Mutorf7_2_A**	AATGATATCGGCATCAACAGCGACTTCTC
**Mutorf9_1_B**	AGAACTTGTGGCGAACTGGGACATC

Figure S1: Generation of mutant MARPPIII. (A) Scheme representing the insertion of pBBSKTT1701 into *S. argillaceus* chromosome. WT, wild type strain; *tsr*, thiostrepton resistance gene; (B) PCR analysis of MARPPIII mutant. PCR products from the wild type (WT) strain and from MARPPIII mutant (PIII), using oligonucleotides M13FW/SA1701FW. λ, Pst-digested Lambda DNA

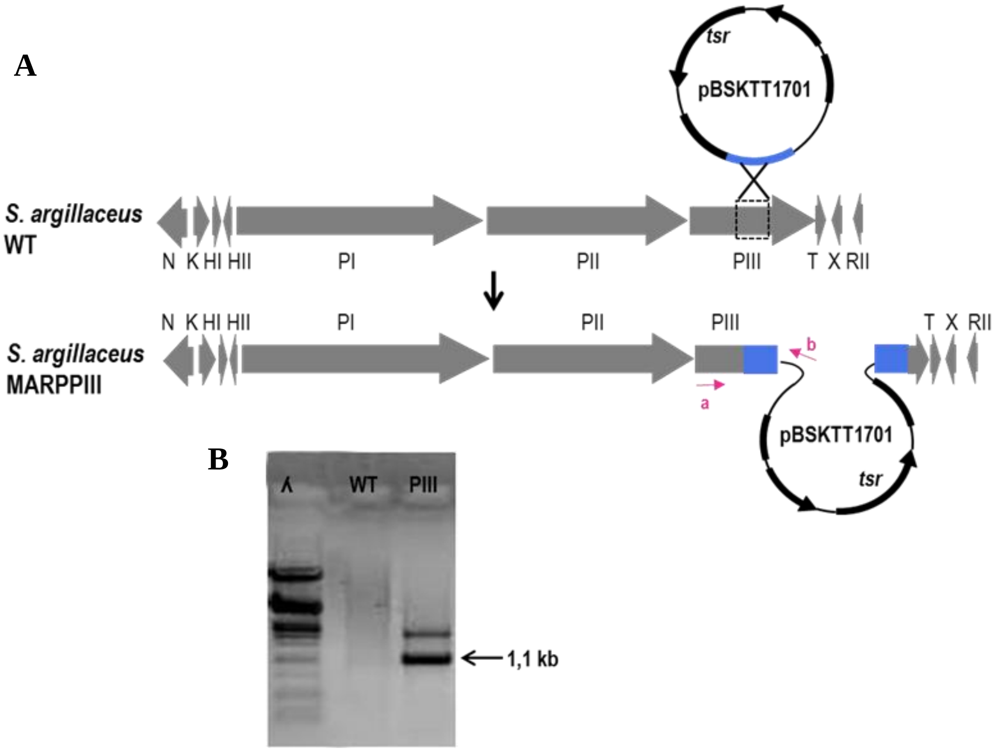

Figure S2: Generation of mutant MARPRII. (A) Scheme representing the replacement event for generation of mutant MARPRII. WT, wild type strain; *aac(3)IV*, apramycin resistance gene; (B) PCR analysis of MARPRII mutant. PCR products from the wild type (WT) strain and from MARPRII mutant, using oligonucleotides MutTetRcompr_A/MutTetRcompr_B. λ, Pst-digested Lambda DNA

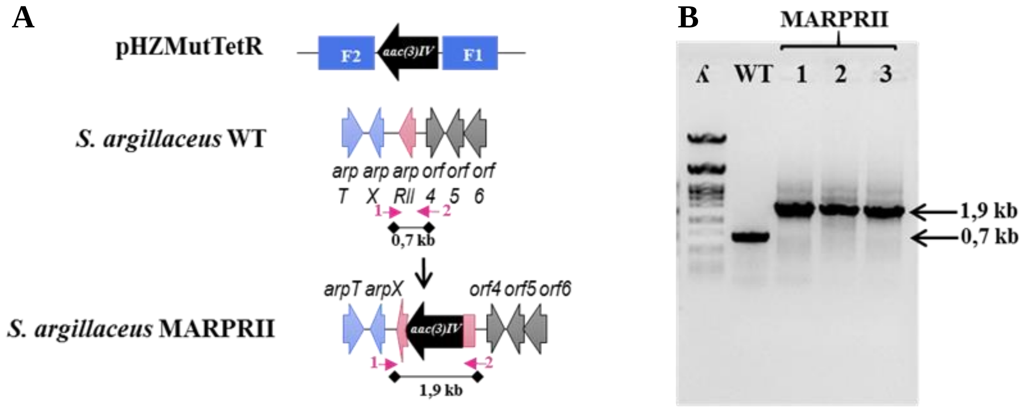

Figure S3: Experimental (A) and simulated (B) HRMS spectra for a molecular formula of $C_{17}H_{19}N_2O_3S$. The agreement between these data is due to the isotope pattern of sulfur containing molecules.

A

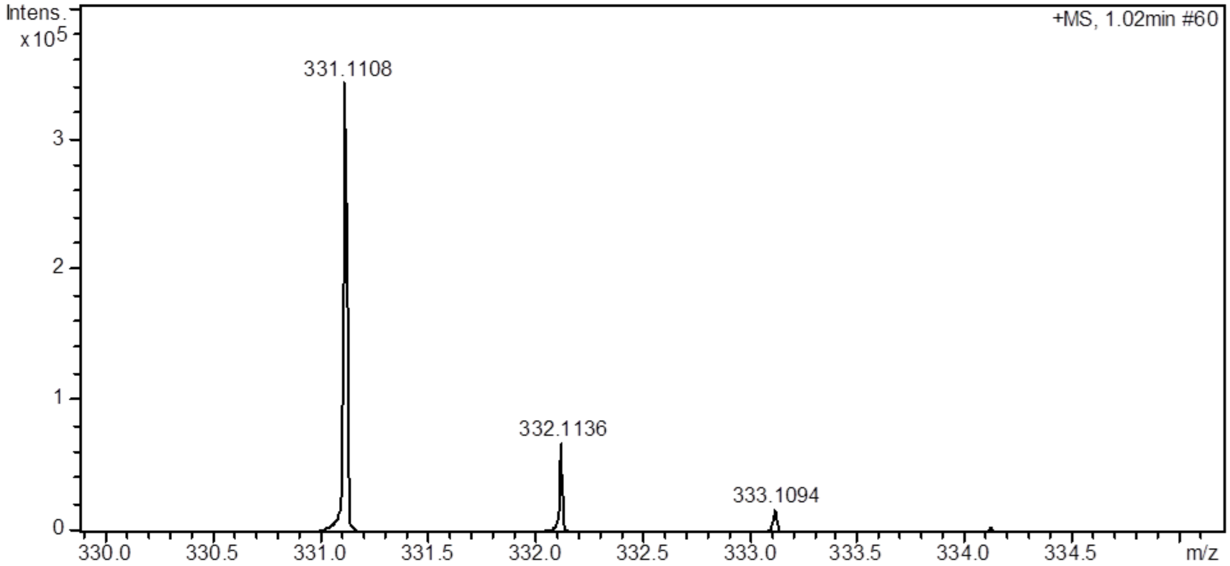

B

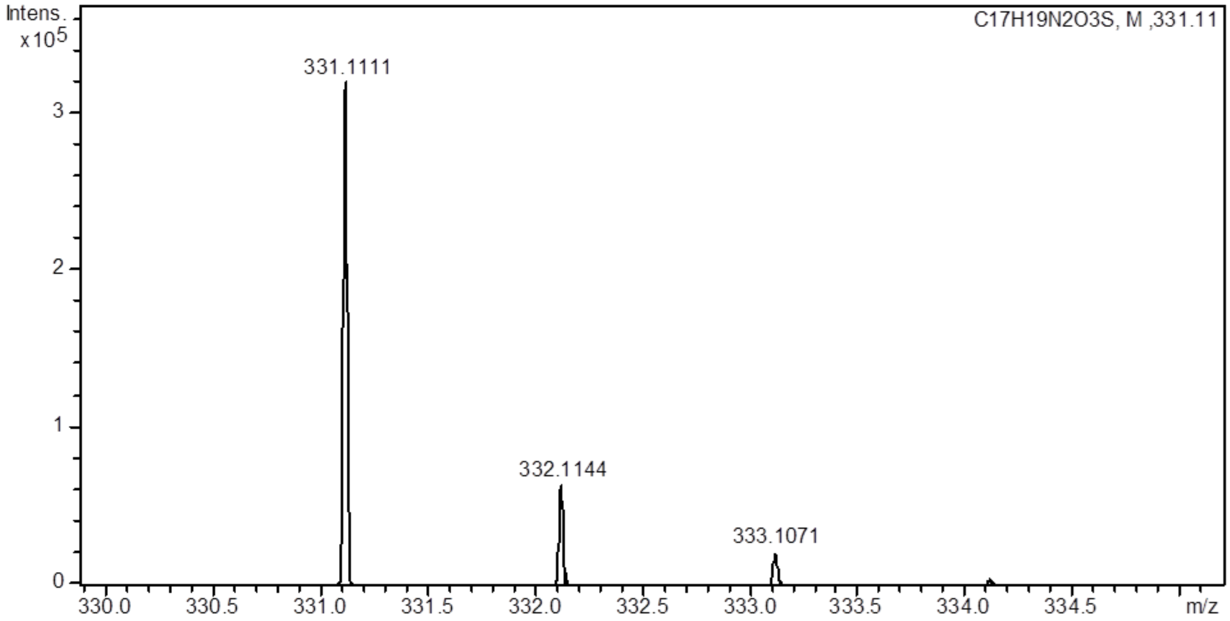

Figure S4: ^{1}H-NMR spectrum of argimycin PI (CD_3OD, 500 MHz). **A**: overall chart. **B**: expansion of the olefinic region. **C**: expansion of the aliphatic region.

A

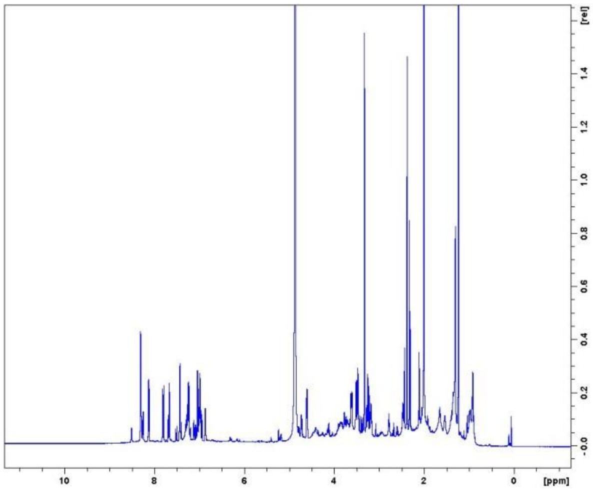

B

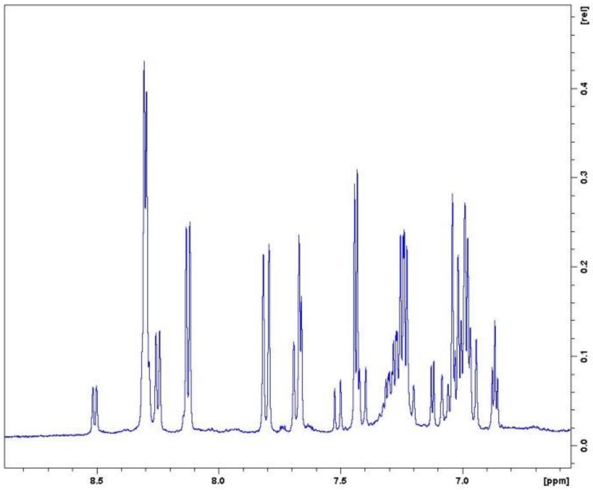

C

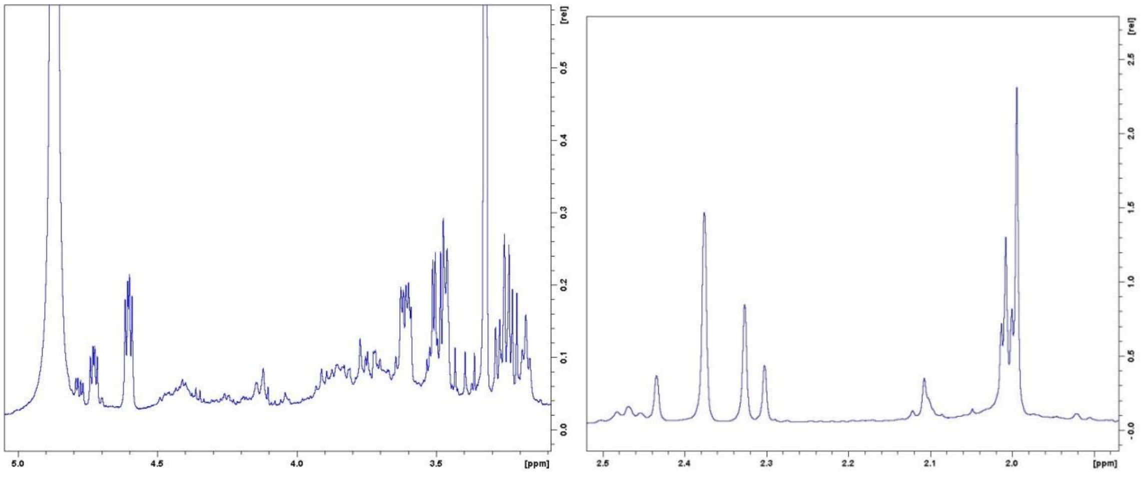

Figure S5: HSQC spectrum of argimycin PI.

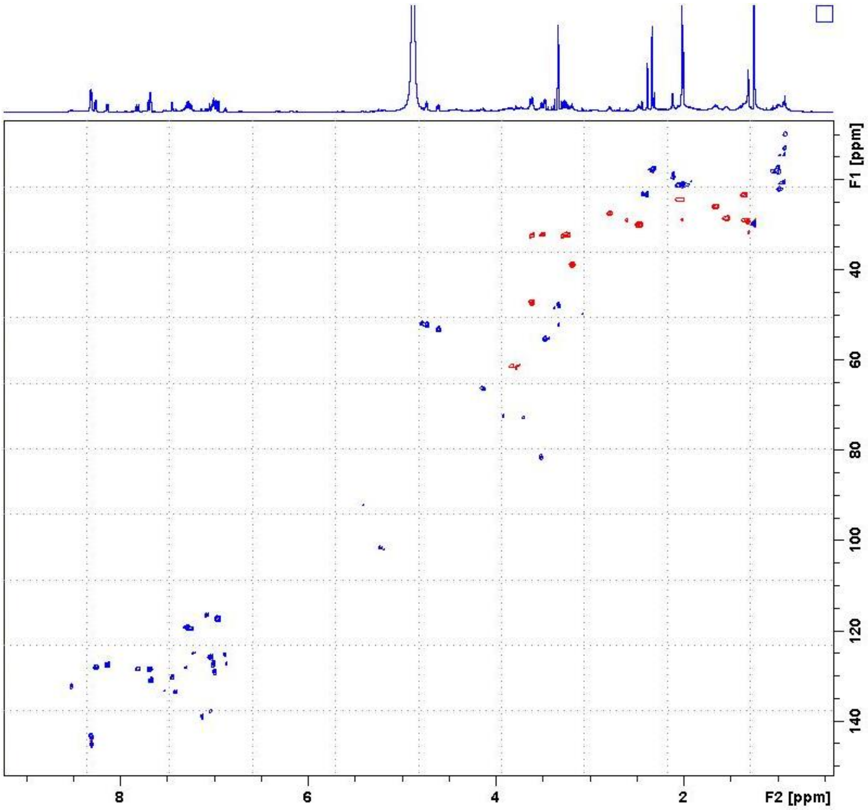

Figure S6: ^{15}N-HMBC spectrum of argimycin PI.

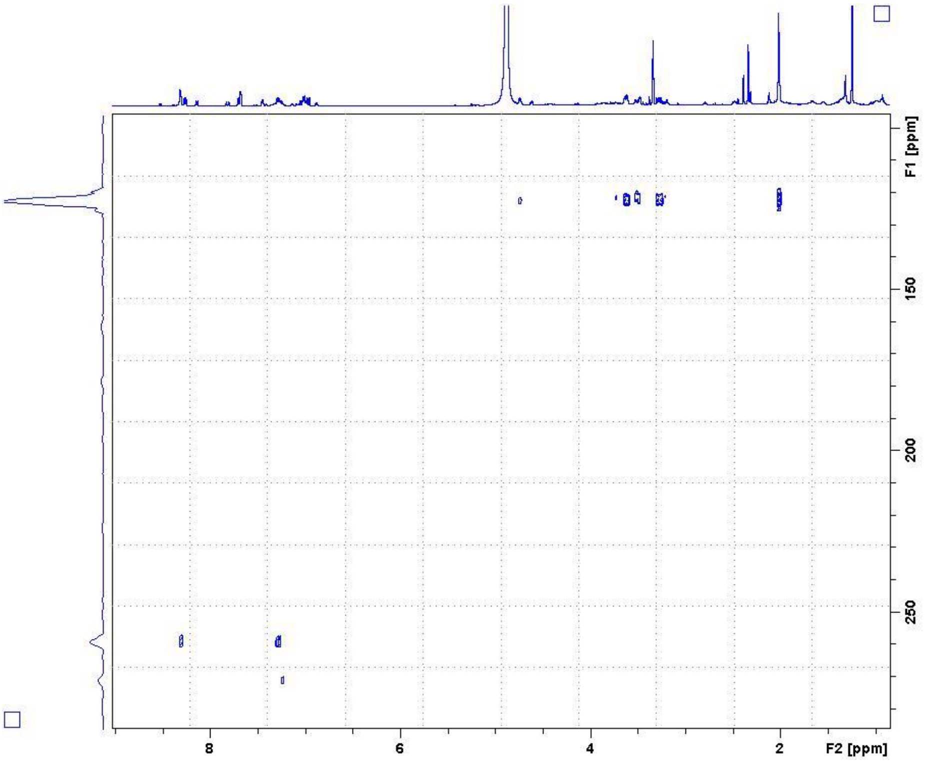

Figure S7: NOESY spectrum of argimycin PI.

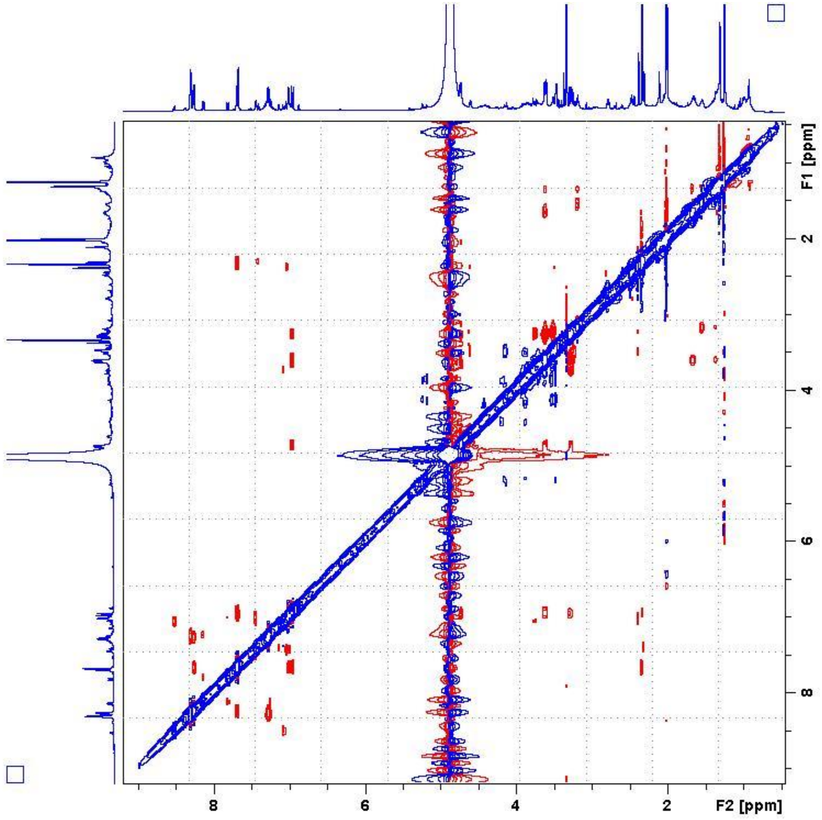

Figure S8: ^{1}H-NMR spectrum of argimycin PII (CD_3OD, 500 MHz). **A**: overall chart. **B**: expansion of the olefinic region. **C**: expansion of the aliphatic region.

A

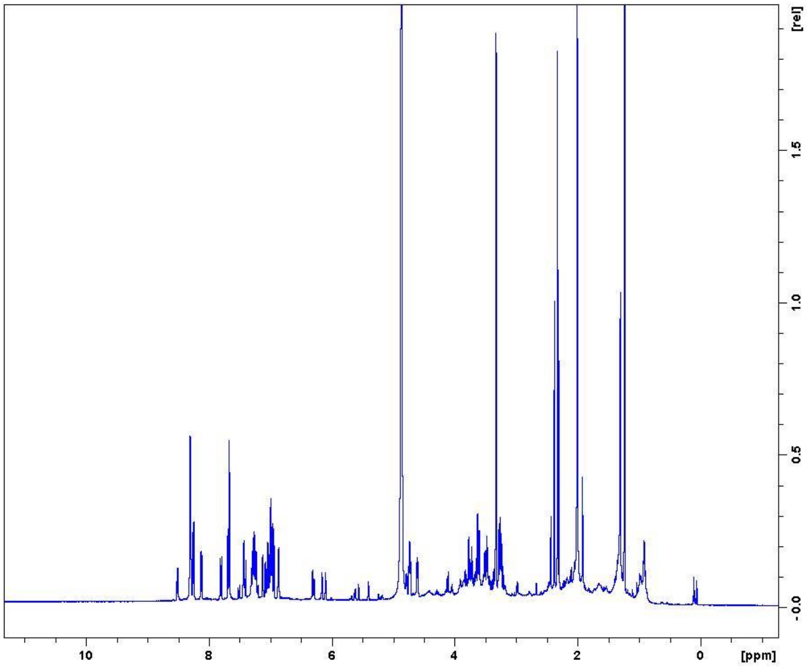

B

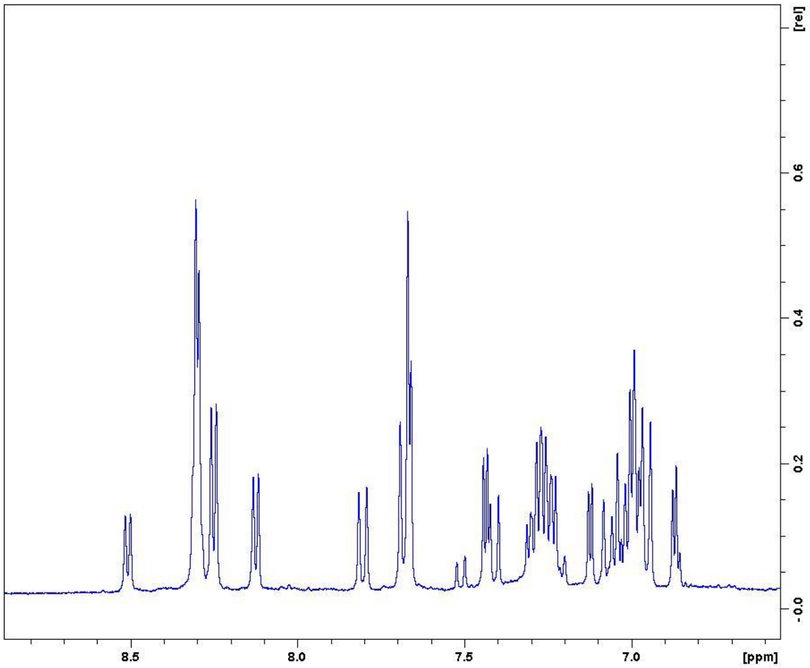
[rel]
0.6
0.4
0.2
-0.0
8.5
8.0
7.5
7.0
[ppm]

C

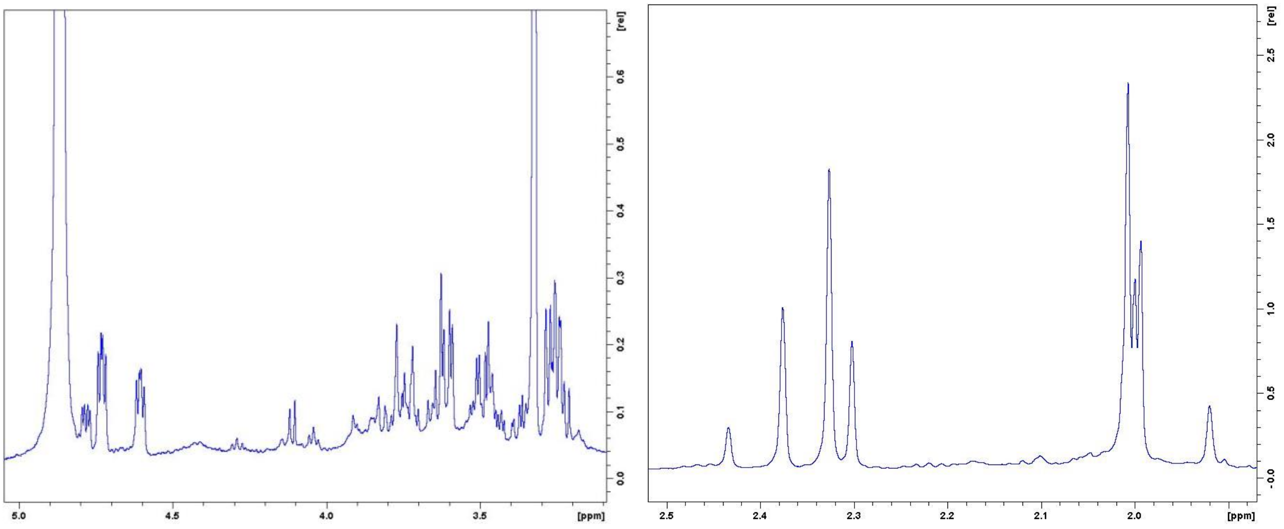
[rel]
0.6
0.5
0.4
0.3
0.2
0.1
0.0
5.0
4.5
4.0
3.5
[ppm]
[rel]
2.5
2.0
1.5
1.0
0.5
-0.0
2.5
2.4
2.3
2.2
2.1
2.0
[ppm]

Figure S9: HSQC spectrum of argimycin PII.

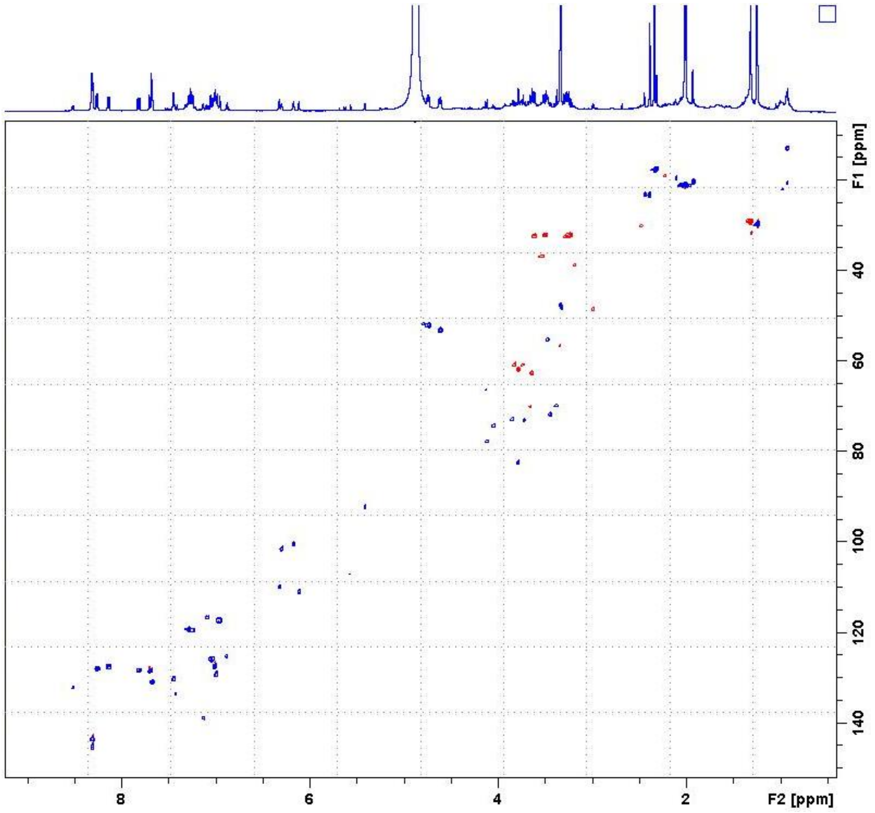

Figure S10: HMBC spectrum of argimycin PII.

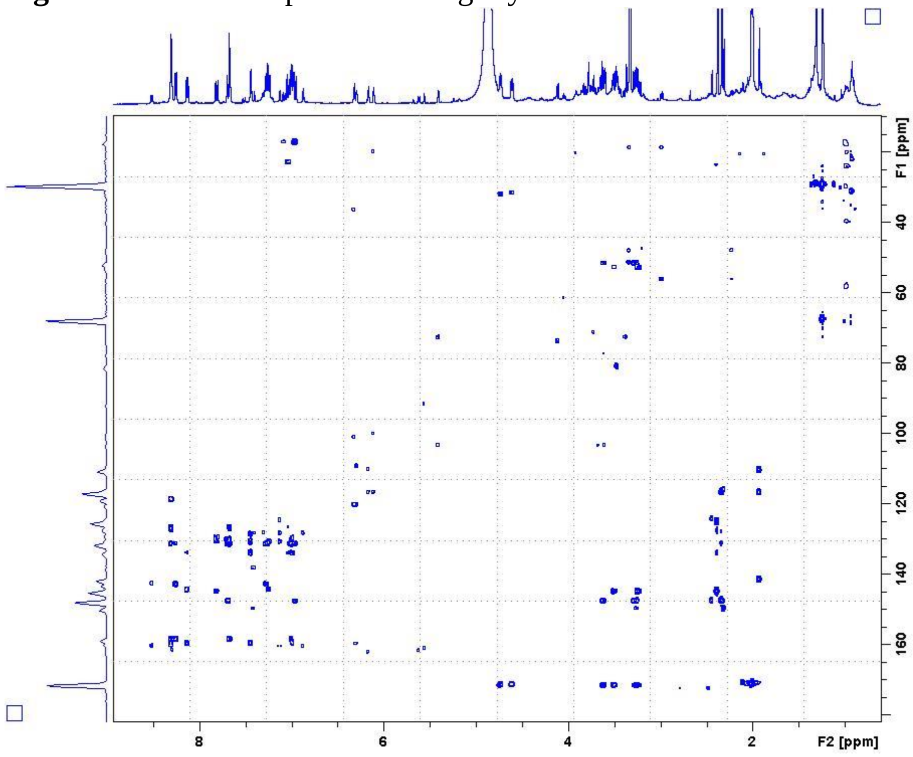

Figure S11: COSY spectrum of argimycin PII.

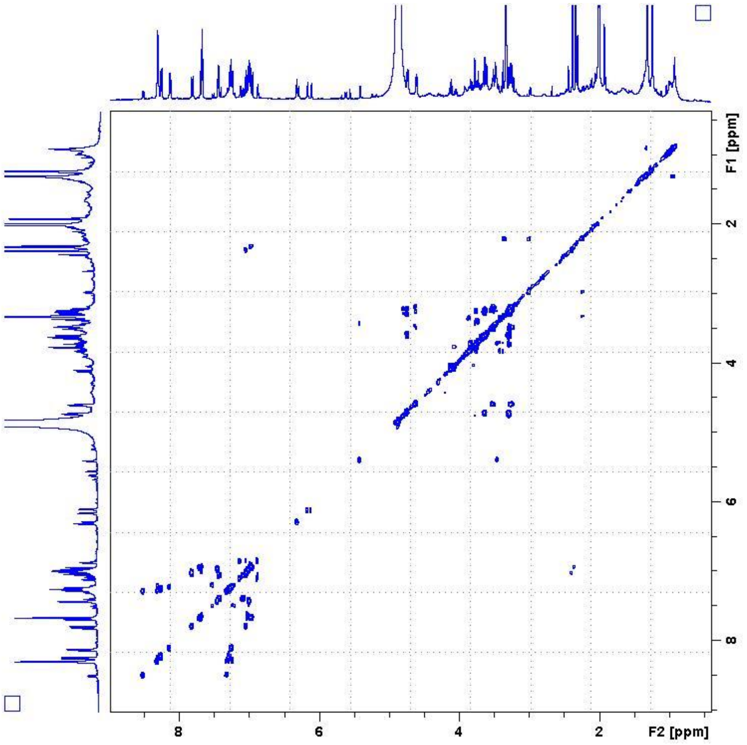

Figure S12: argimycin PI signal assignment. Left: ^{1}H- and ^{13}C-NMR signals (^{1}H blue, ^{13}C red, ^{15}N green). Right: observed correlations from the NOESY spectrum.

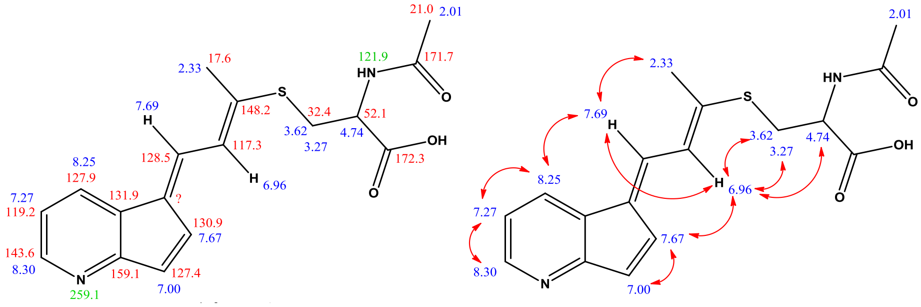

Figure S13: argimycin PII, observed key correlations from the NOESY spectrum enable the determination of the double bond stereochemistry**.**

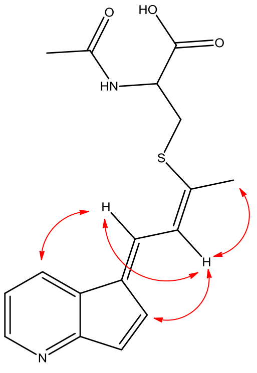

Figure S14: Mechanism of decarboxylation (argimycin PI shown) explaining the main MS fragment observed both in ESI and in MS/MS experiments. Fragmentation of the parent ion under ESI- provides $[M\text{-}H]^-$ (*m/z* 329), indicating an acid proton from the carboxylic acid. Additionally another fragment $C_{12}H_{10}NS^-$ (*m/z* 200) is observed from the parent ion, which is explained by the mechanism of decarboxylation shown below.

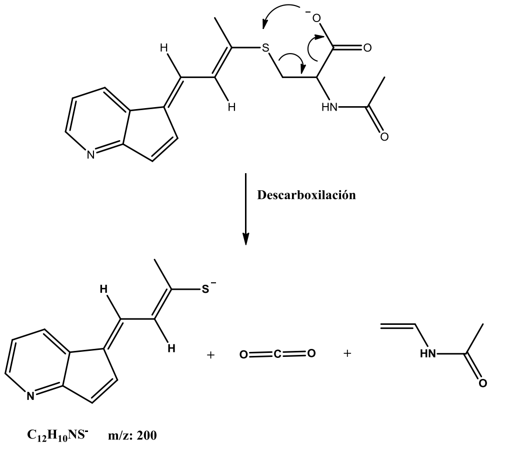

Figure S15: LC/MS analysis of desulfurization/reduction product of argimycin PI/PII subjected to Marfey's analysis.

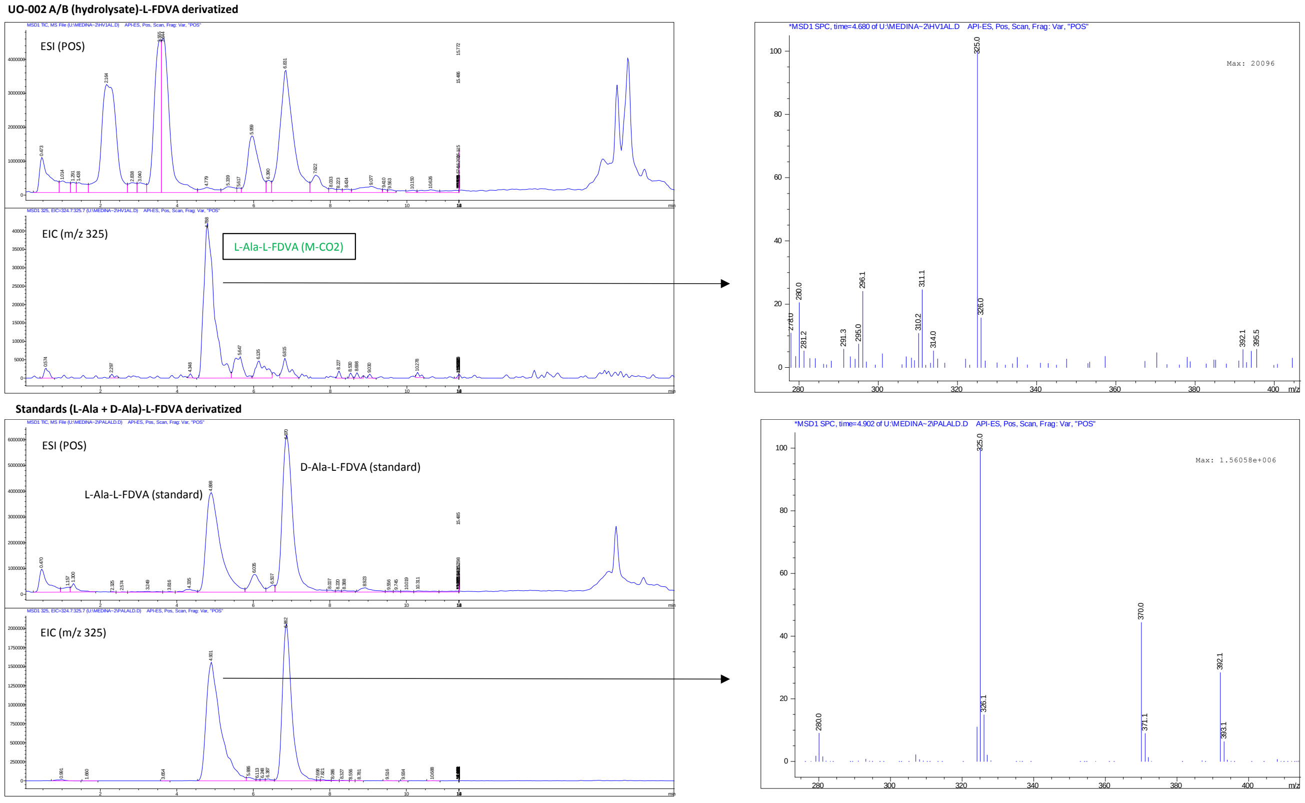

Figure S16: Comparison of the ^{1}H-NMR spectra of argimycin PV (red) and argimycin PIV (blue). **A**: overall chart. **B**: detail of the olefinic region. **C**: expansion of the aliphatic region.

A

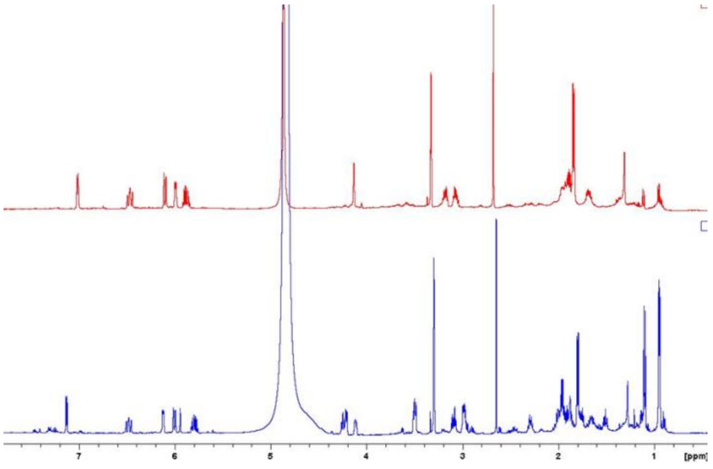

B

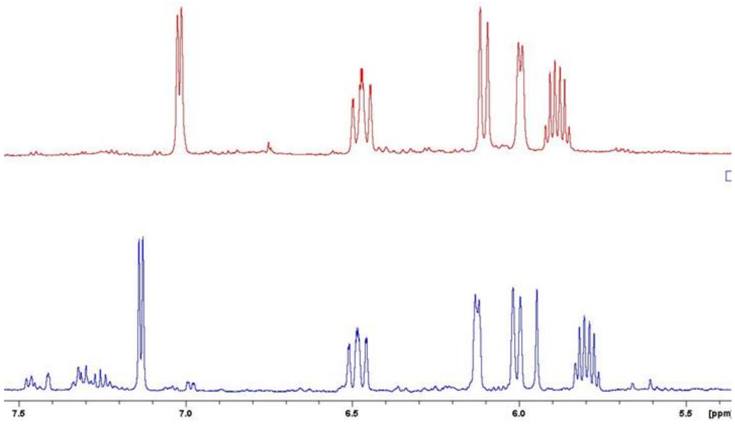

C

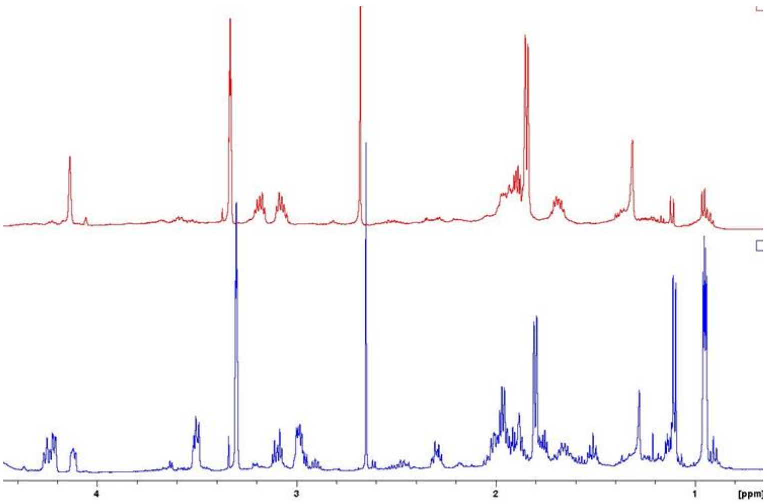

Figure S17: HSQC spectrum of the aliphatic region of argimycin PVI showing the identification of the assigned signals.

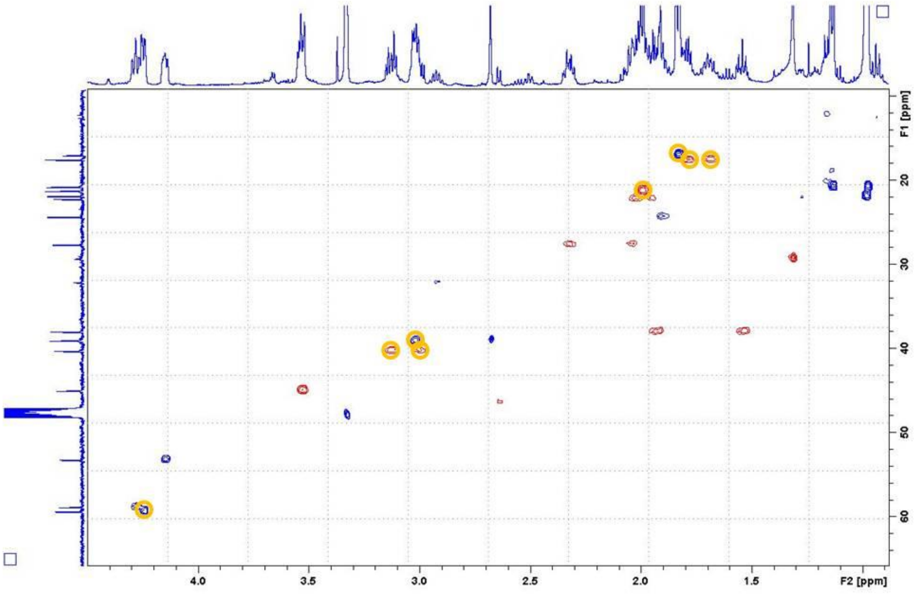

Figure S18: ^{1}H-NMR spectrum of argimycin PIV acquired in CD_3OD (blue) and in DMSO-d_6 (red) at 24°C.

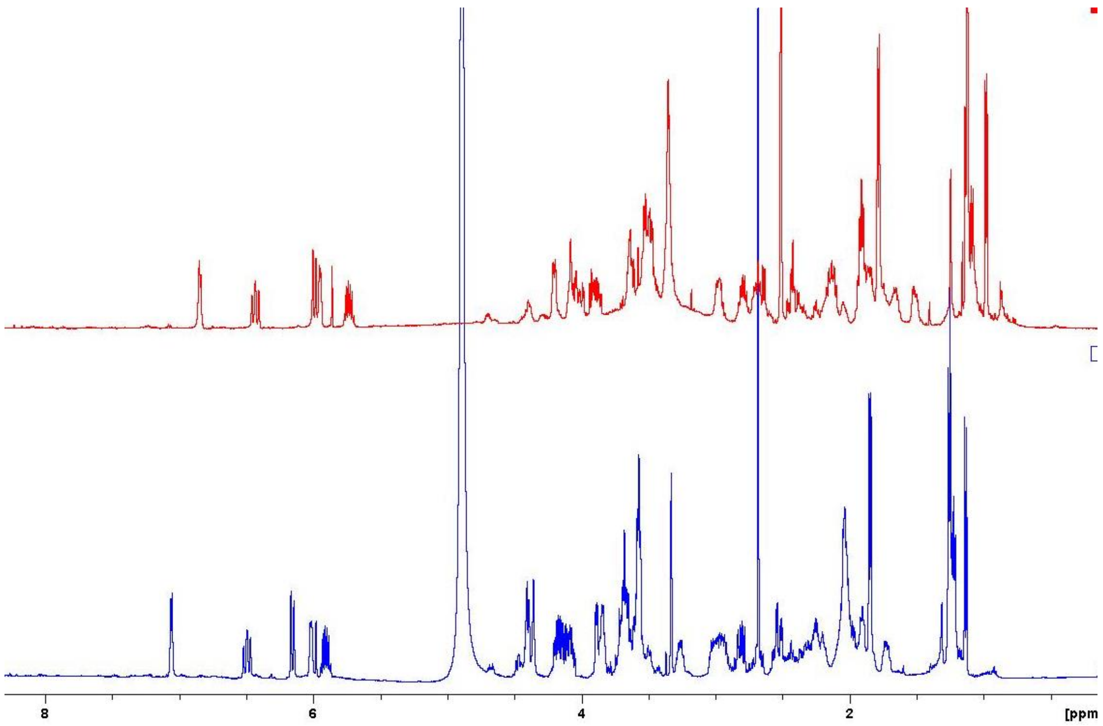

Figure S19: Comparison of the ^{1}H-NMR spectra of argimycin PIV (blue), argimycin PV (red) and argimycin PVI (green). **A**: overall chart. **B**: expansion of the olefinic region. **C**: expansion of the aliphatic region.

A

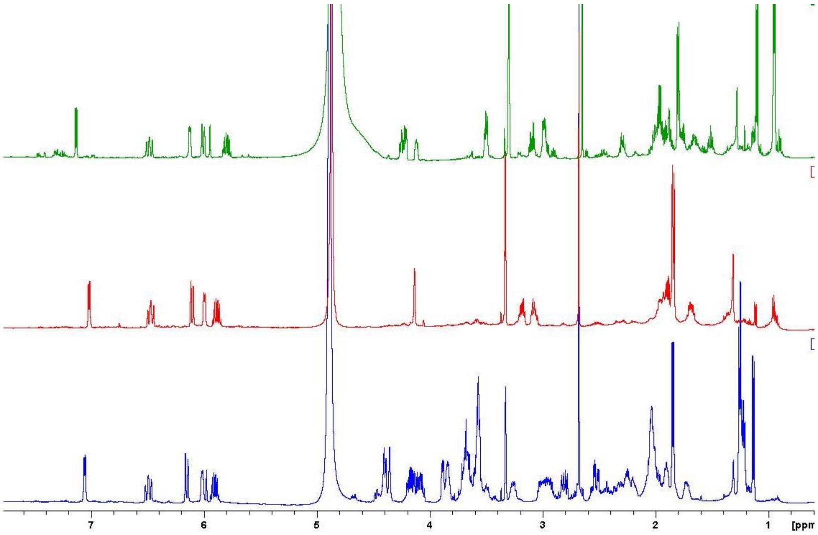

B

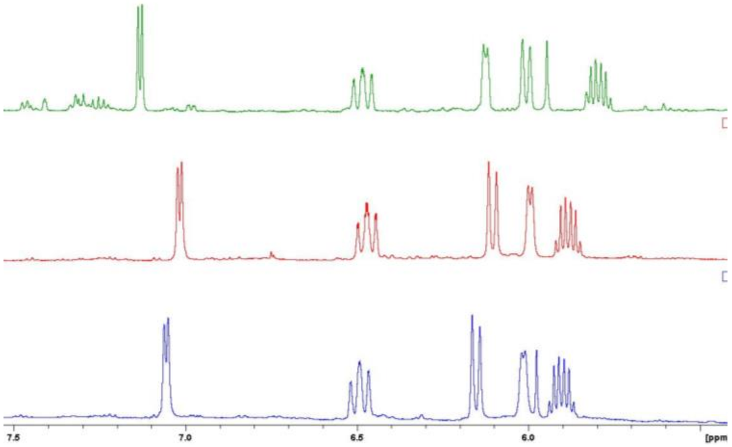

C

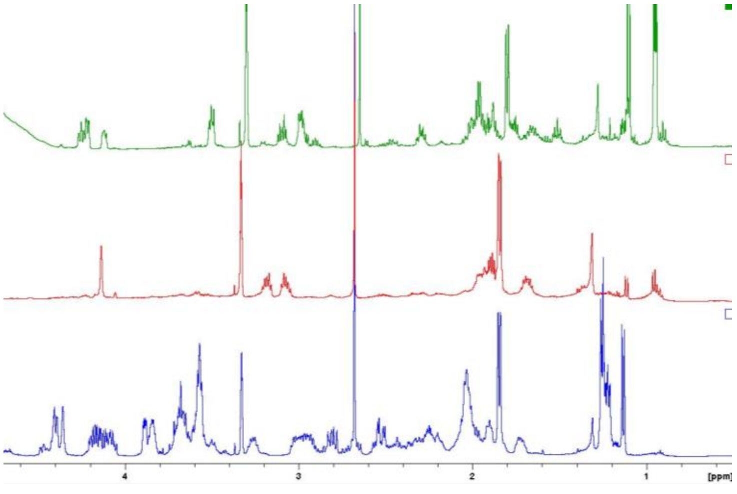

Figure S20: HSQC spectrum of the aliphatic region of argimycin PIV showing the identification of the assigned signals.

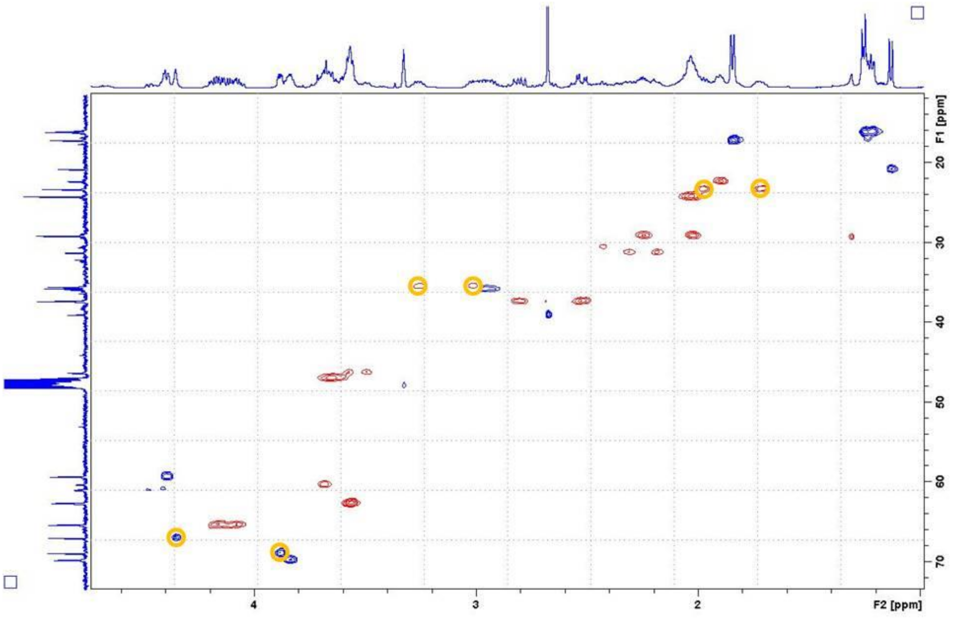

Figure S21: 3D models of argimycin PIV, showing dihedral angles between H4 and H3a and H3b. According to the observed coupling constants, the only compatible model is the one shown in the left (both hydroxyls *cis*).

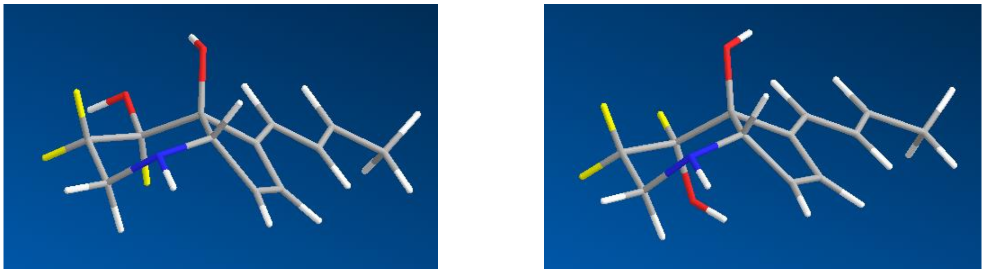

Figure S22: ^{1}H-NMR spectrum of argimycin PIII (nigrifactin) acquired in CD_3OD (blue) and in DMSO-d_6 (red) at 24°C.

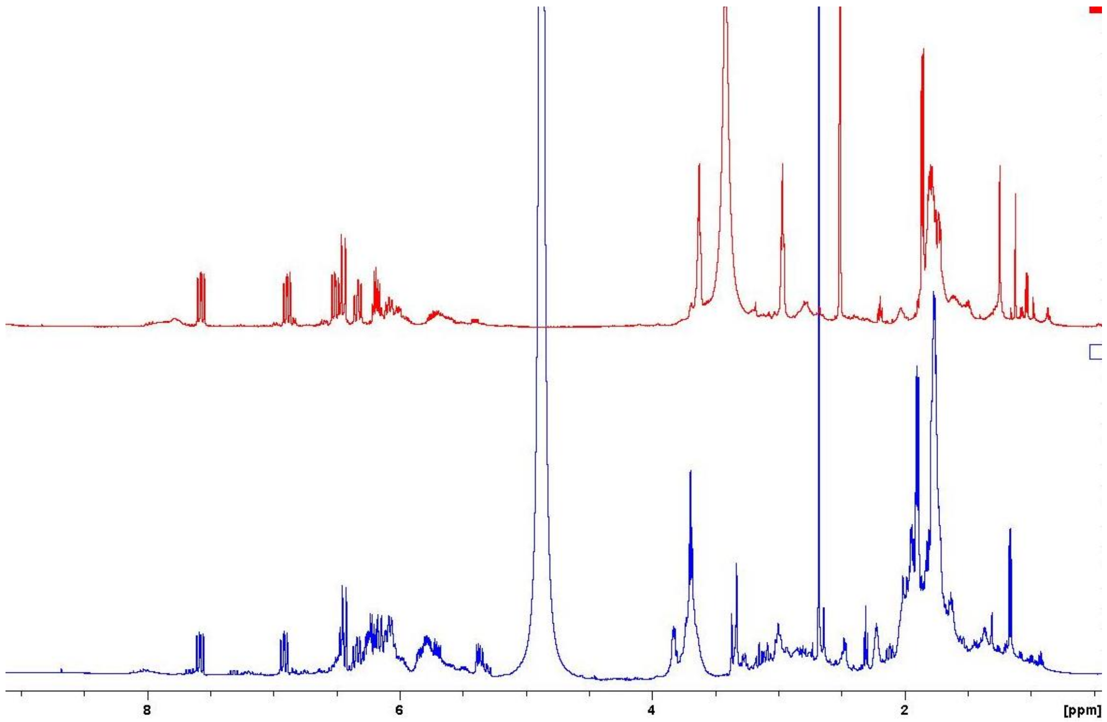

Figure S23: Comparison of the ^{1}H-NMR spectra of argimycin PIII (nigrifactin) (blue), argimycin PIV (red), argimycin PV (green) and argimycin PVI (purple). **A**: overall chart. **B**: expansion of the olefinic region. **C**: expansion of the aliphatic region.

A

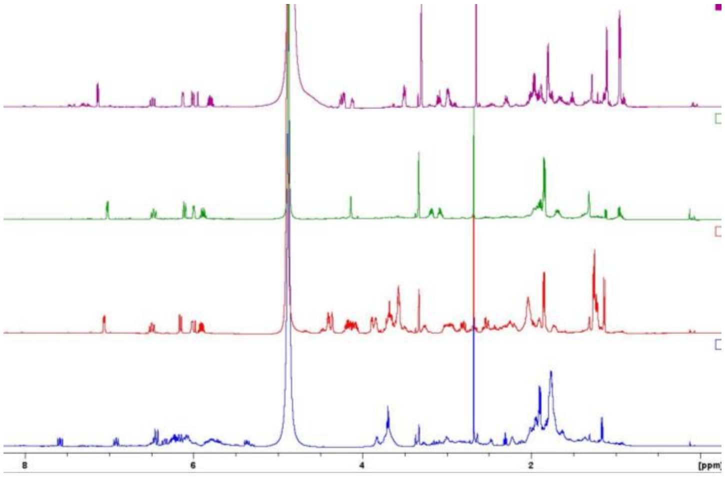

B

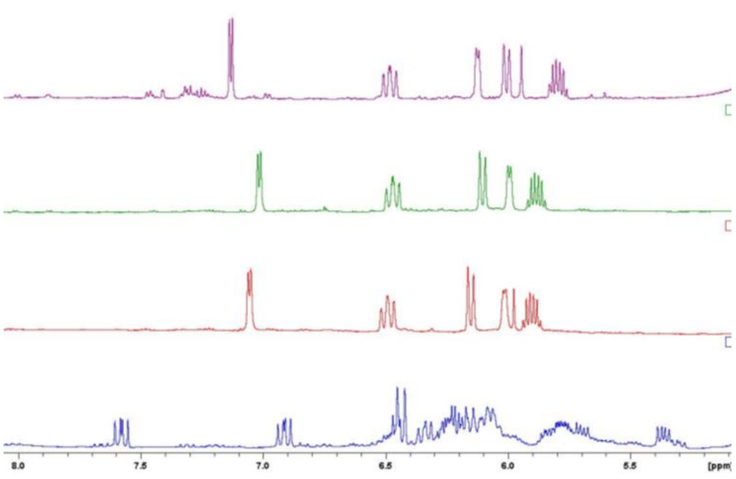

C

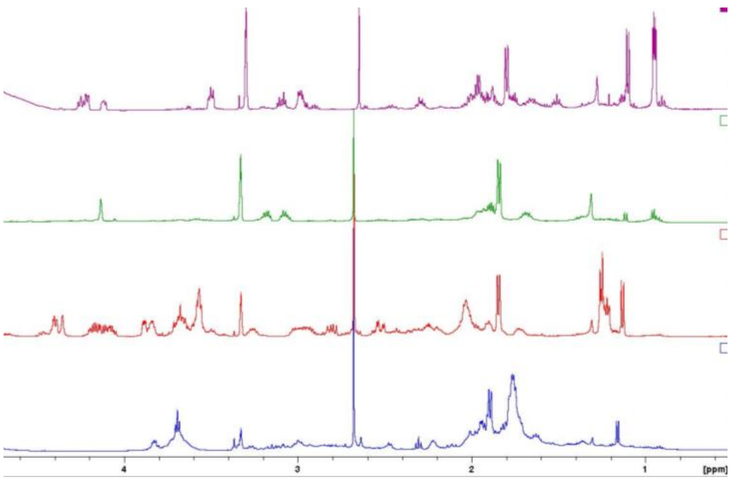

Figure S24: ^{1}H-NMR spectrum of argimycin PIX (CD_3OD, 500 MHz). **A**: overall chart. **B**: expansion of the olefinic region. **C**: expansion of the aliphatic region.

A

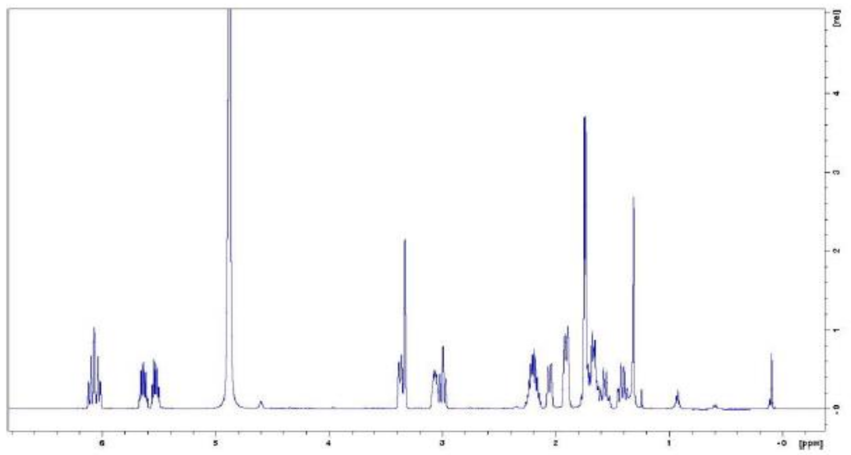

B

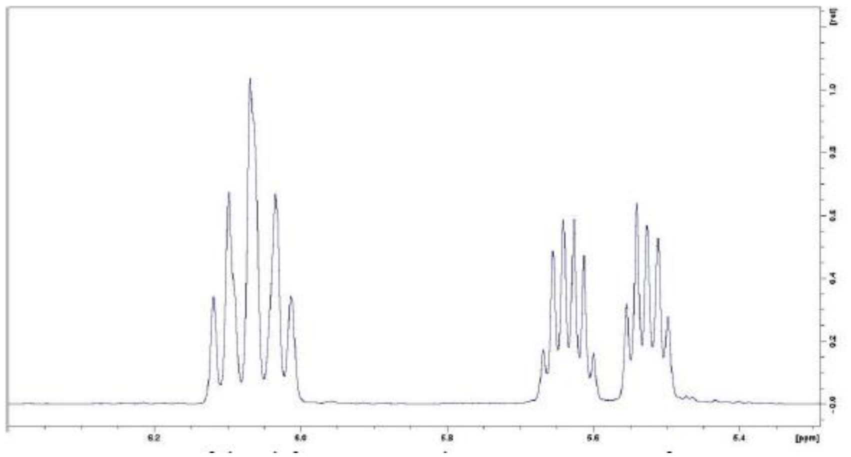

C

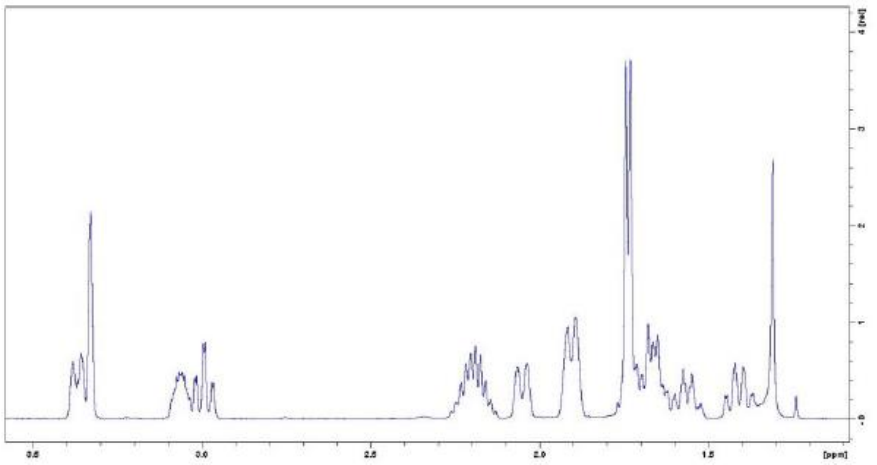

Figure S25: HSQC spectrum of argimycin PIX.

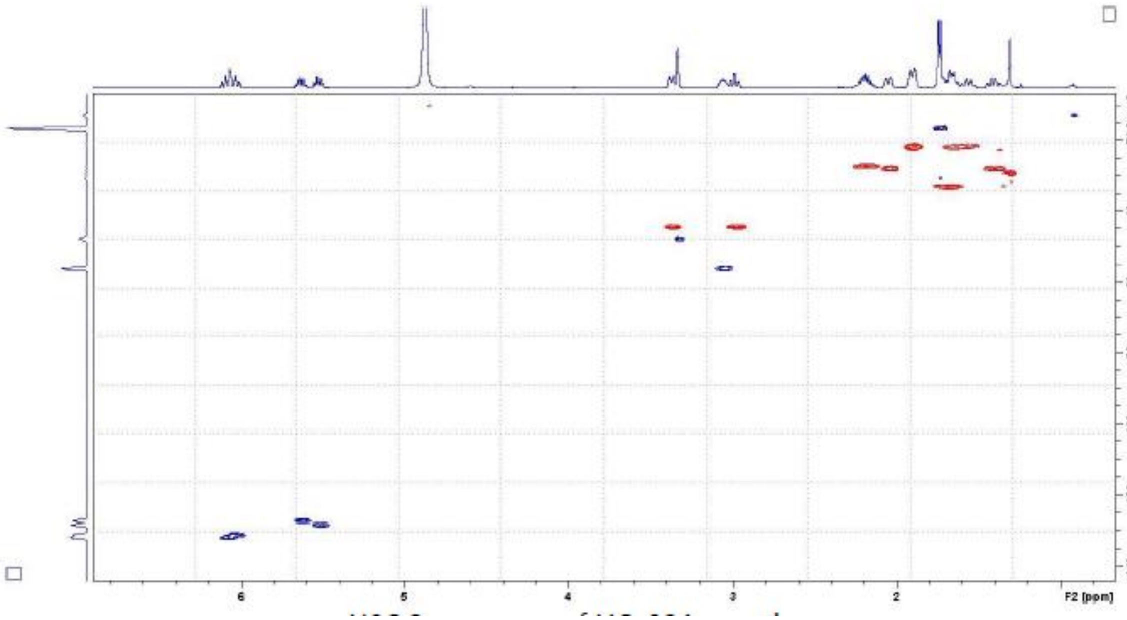

Figure S26: Generation of mutant MORF3. (A) Scheme representing the replacement event for generation of mutant MORF3. WT, wild type strain; *aac(3)IV*, apramycin resistance gene; (B) PCR analysis of MORF3 mutant. PCR products from the wild type (WT) strain and from MORF3 mutant, using oligonucleotides Orf3_A/Orf3_B. λ, Pst-digested Lambda DNA

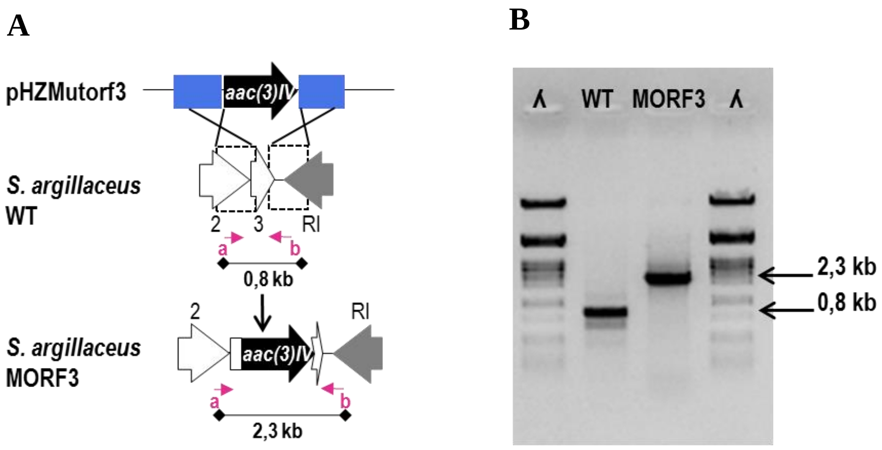

Figure S27: Generation of mutant MARPRI. (A) Scheme representing the replacement event for generation of mutant MARPRI. WT, wild type strain; *aac(3)IV*, apramycin resistance gene; (B) PCR analysis of MARPRI mutant. PCR products from the wild type (WT) strain and from MARPRI mutant (isolates 1 and 2), using oligonucleotides 378orf1Abis/378orf1B. λ, Pst-digested Lambda DNA

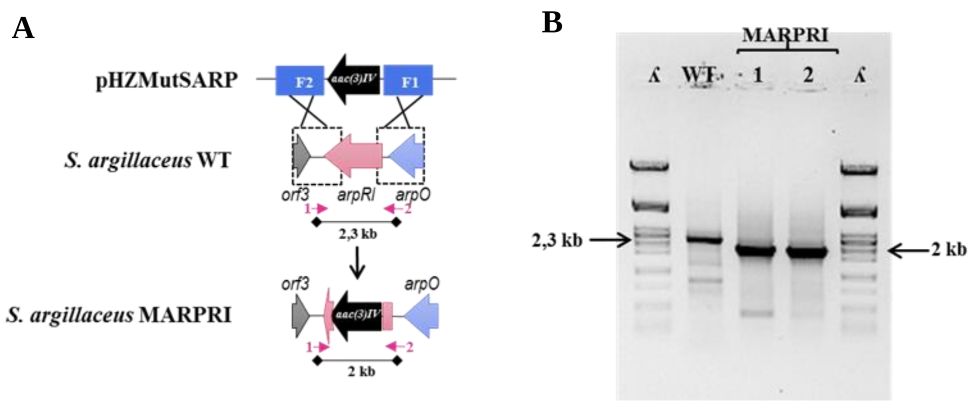

Figure S28: Generation of mutant DORF19-21. (A) Scheme representing the replacement event for generation of mutant DORF19-21. WT, wild type strain; *aac(3)IV*, apramycin resistance gene; (B) PCR analysis of DORF19-21 mutant. PCR products from the wild type (WT) strain and from DORF19-21 mutant (isolates 1-3), using oligonucleotides Del2_A_comp/ApraC_rv (Frag 1) and Del2_B_comp/ApraC_fw (Frag 2). λ, Pst-digested Lambda DNA

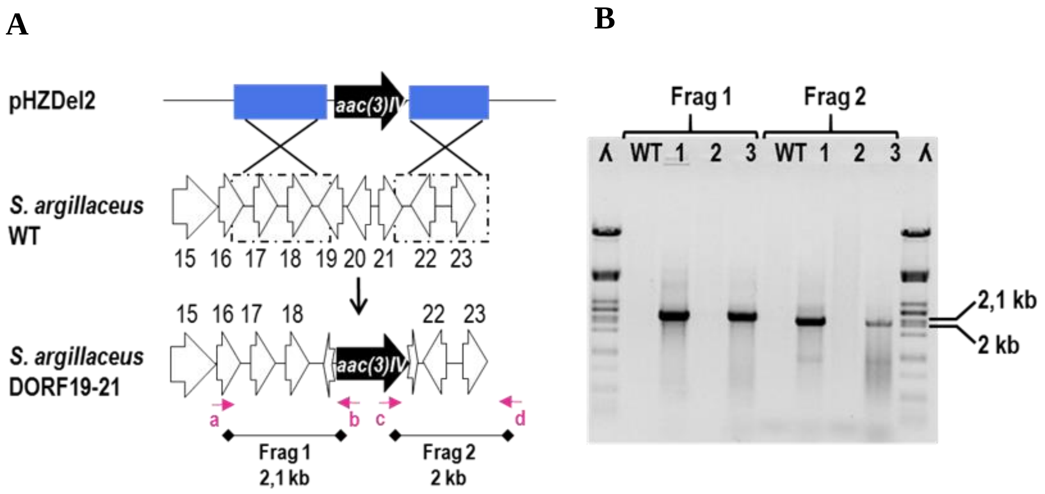

Figure S29: Generation of mutant DORF11-16. (A) Scheme representing the replacement event for generation of mutant DORF11-16. WT, wild type strain; *aac(3)IV*, apramycin resistance gene; (B) PCR analysis of DORF11-16 mutant. PCR products from the wild type (WT) strain and from DORF11-16 mutant (isolates 1, 2), using oligonucleotides Del3_A_comp/ApraC_rv (Frag 1) and Del3_B_comp/ApraC_fw (Frag 2). λ, Pst-digested Lambda DNA

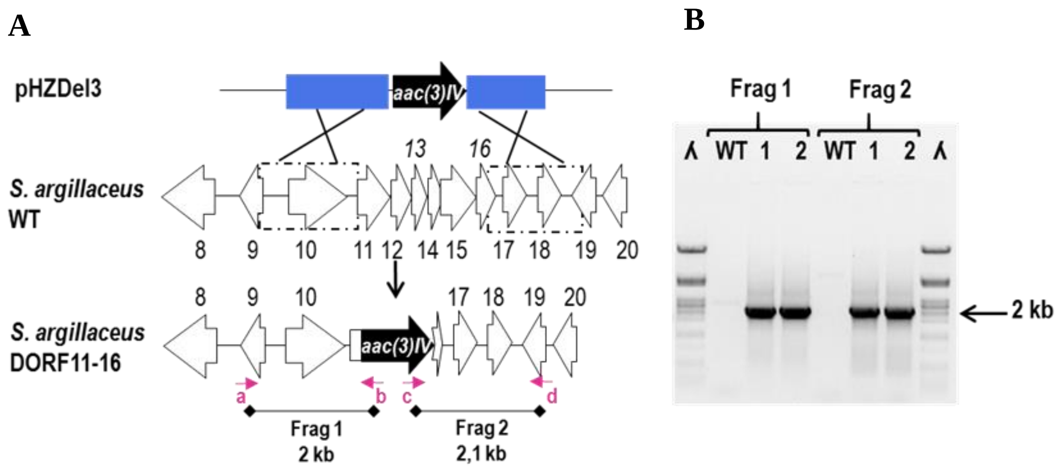

Figure S30: Generation of mutant MORF9. (A) Scheme representing the replacement event for generation of mutant MORF9. WT, wild type strain; *aac(3)IV*, apramycin resistance gene; (B) PCR analysis of MORF9 mutant. PCR products from the wild type (WT) strain and from MORF9 mutant (isolates 1-4), using oligonucleotides MutNAcTrC_A/MutNAcTrC_B. λ, Pst-digested Lambda DNA

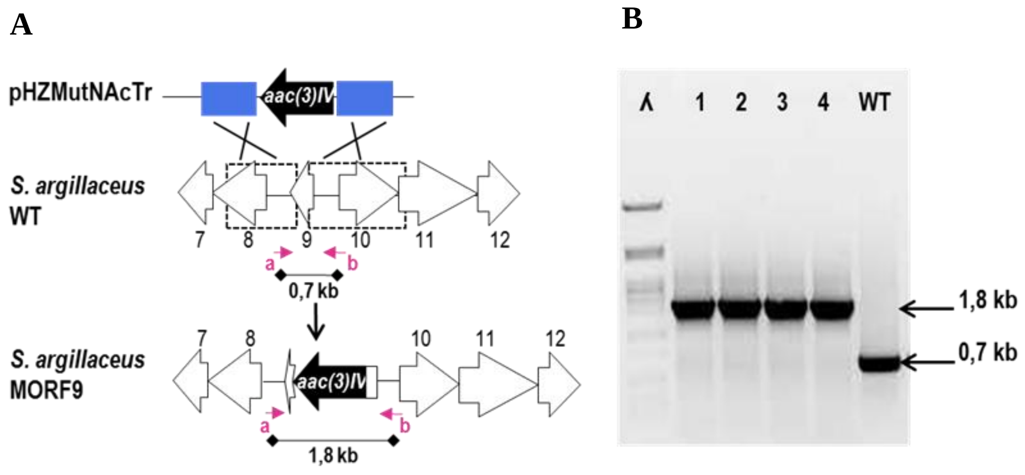

Figure S31: Generation of mutant DORF5-7. (A) Scheme representing the replacement event for generation of mutant DORF5-7. WT, wild type strain; *aac(3)IV*, apramycin resistance gene; (B) PCR analysis of DORF5-7 mutant. PCR products from the wild type (WT) strain and from DORF5-7 mutant (isolates 1, 2), using oligonucleotides MutNAcTr2_A/ApraC_rv (Frag 1) and 1701orf4RT_A/ApraC_fw (Frag 2). λ, Pst-digested Lambda DNA

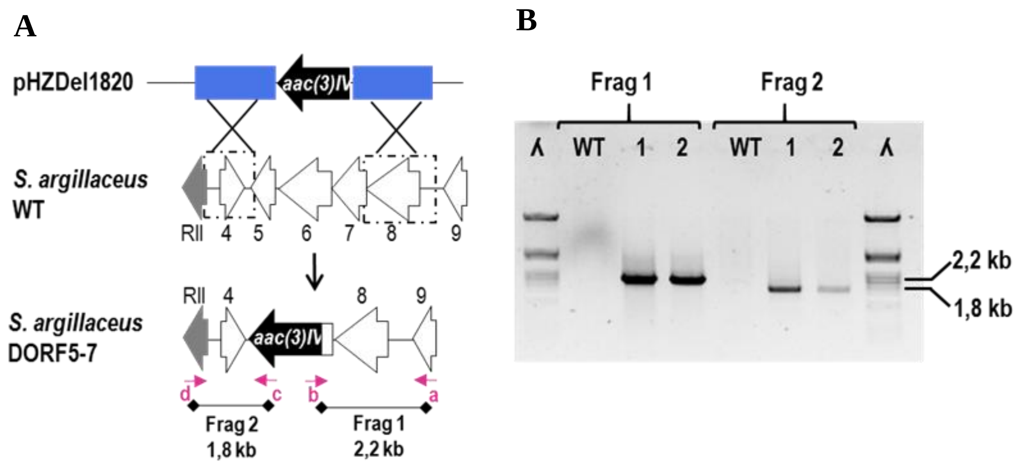

Figure S32: Generation of mutant MORF4. (A) Scheme representing the replacement event for generation of mutant MORF4. WT, wild type strain; *aac(3)IV*, apramycin resistance gene; (B) PCR analysis of MORF4 mutant. PCR products from the wild type (WT) strain and from MORF4 mutant (isolates 1, 2), using oligonucleotides Del1820_2_A/MutTetR_1_B. λ, Pst-digested Lambda DNA

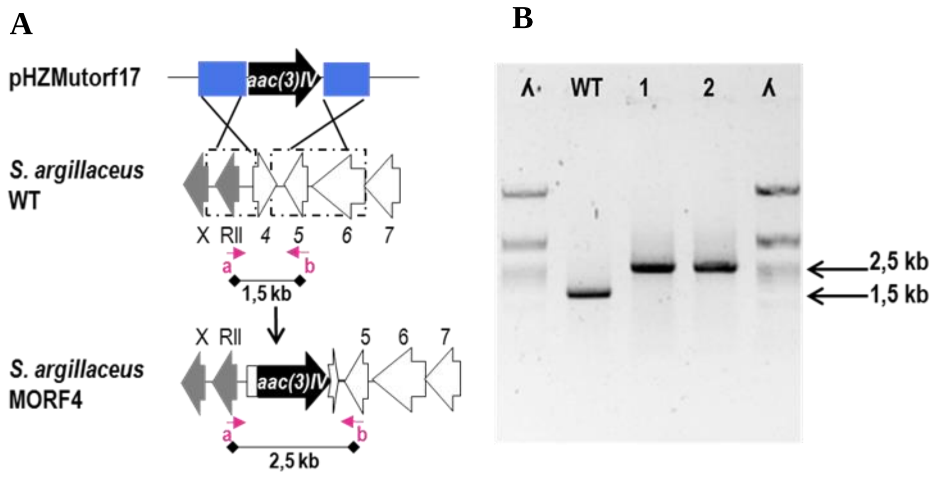

Figure S33: UPLC analysis of butanol extracts from mutants generated in upstream and downstream regions of *arp* cluster. Peaks corresponding to the different argimycins P are indicated as follows: argimycins PI and PII (**a** and **b**); nigrifactin (**c**); argimycin PIV (**d**); argimycin PV (**e**); argimycin PVI (**f**); and argimycin PIX (**g**).

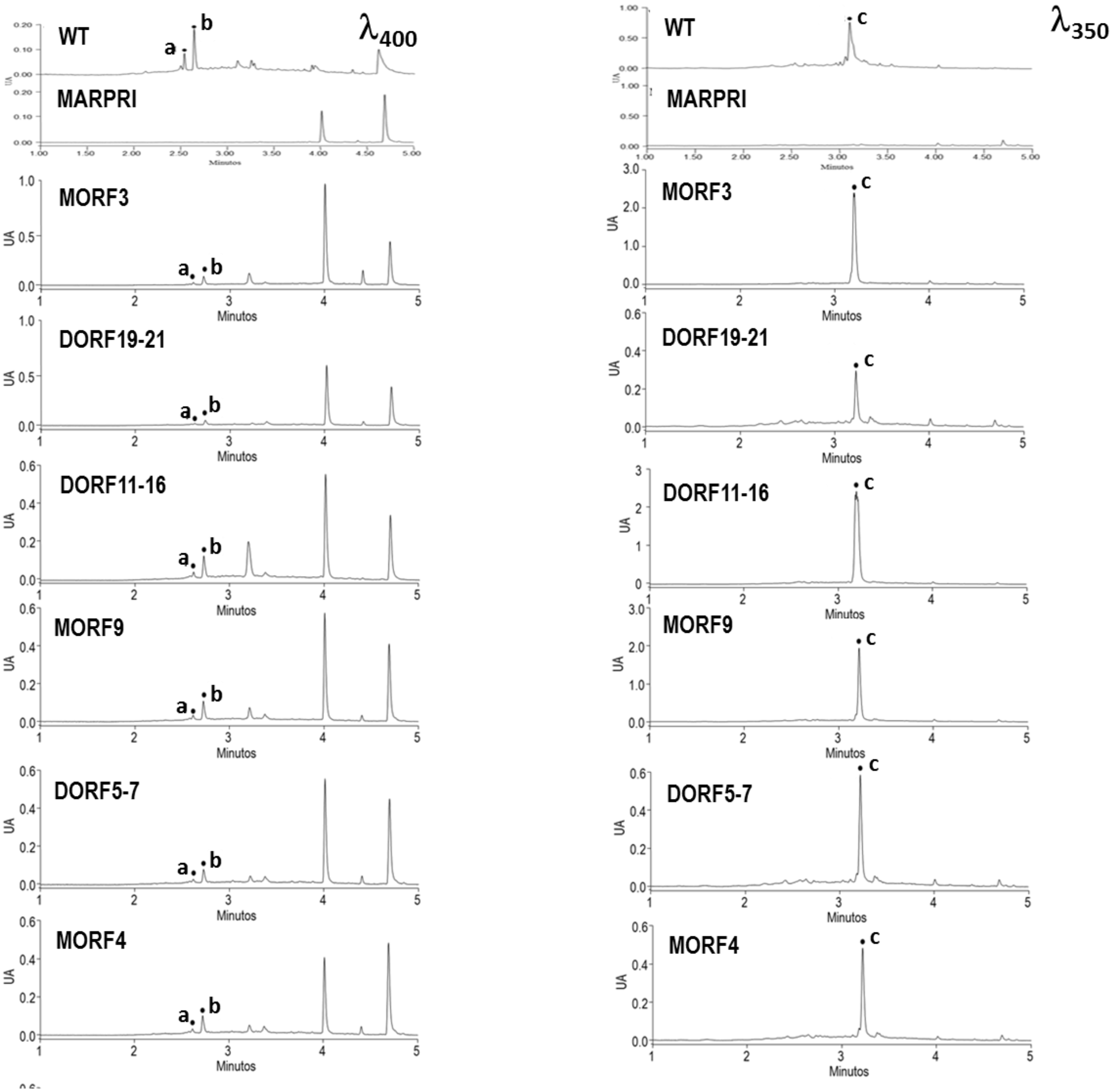

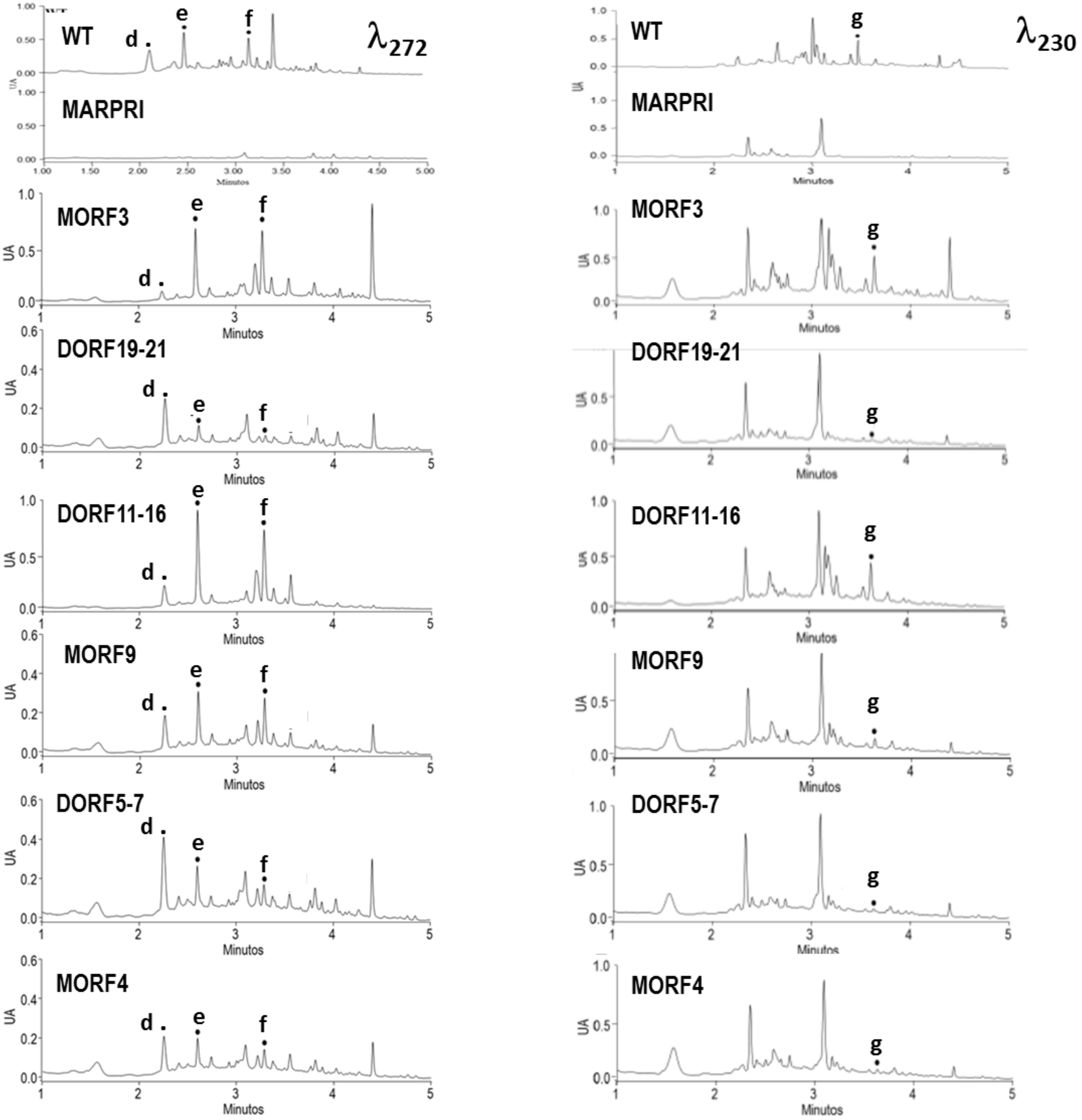
WT
d
e
f
λ_{272}
MARPRI
UA
Minutos
MORF3
DORF19-21
DORF11-16
MORF9
DORF5-7
MORF4
g
λ_{230}

Figure S34: Generation of mutant MARPN. (A) Scheme representing the replacement event for generation of mutant MARPN. WT, wild type strain; *aac(3)IV*, apramycin resistance gene; (B) PCR analysis of MARPN mutant. PCR products from the wild type (WT) strain and from MARPN mutant, using oligonucleotides Mutorf7_2_A and Mutorf9_1_B. λ, Pst-digested Lambda DNA

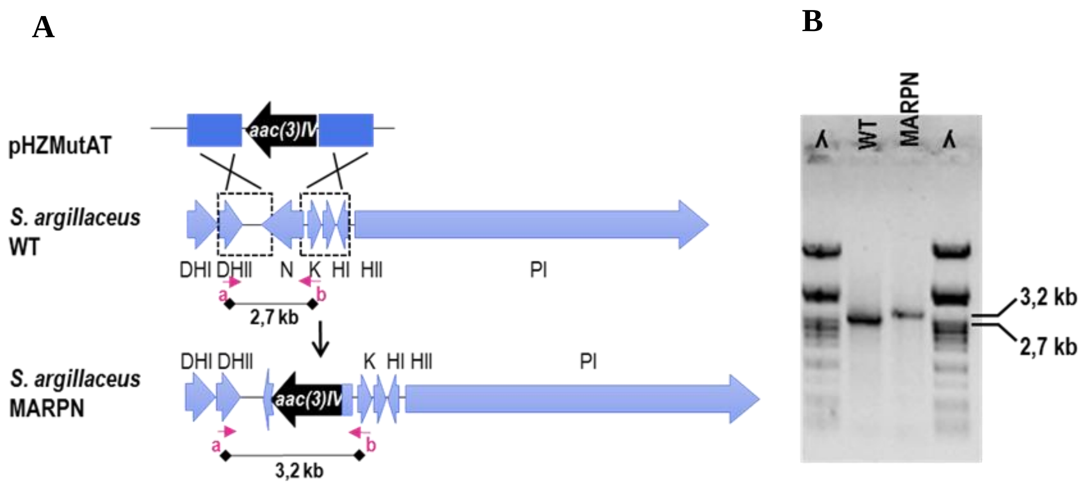

Figure S35: UPLC analysis of butanol extracts from mutant *S. argillaceus* MARPN. Chromatograms are shown at 400 nm (A), 272 nm (B) and 230 nm (C). Peaks corresponding to the different argimycins P are indicated as follows: argimycins PI and PII (**a** and **b**); nigrifactin (**c**); argimycin PIV (**d**); argimycin PV (**e**); argimycin PVI (**f**); and argimycin PIX (**g**).

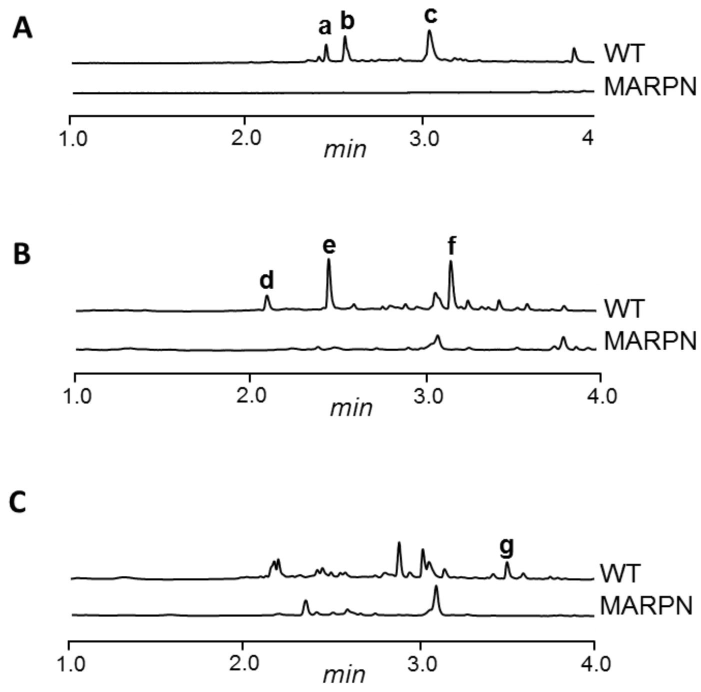

Figure S36: Generation of mutant DARPO-HII. (A) Scheme representing the replacement event for generation of mutant DARPO-HII. WT, wild type strain; *aac(3)IV*, apramycin resistance gene; (B) PCR analysis of DARPO-HII mutant. PCR products from the wild type (WT) strain and from DARPO-HII mutant (isolates 1, 2 and 3), using oligonucleotides Mutorf6_1_A/MutAT1_A. λ, Pst-digested Lambda DNA

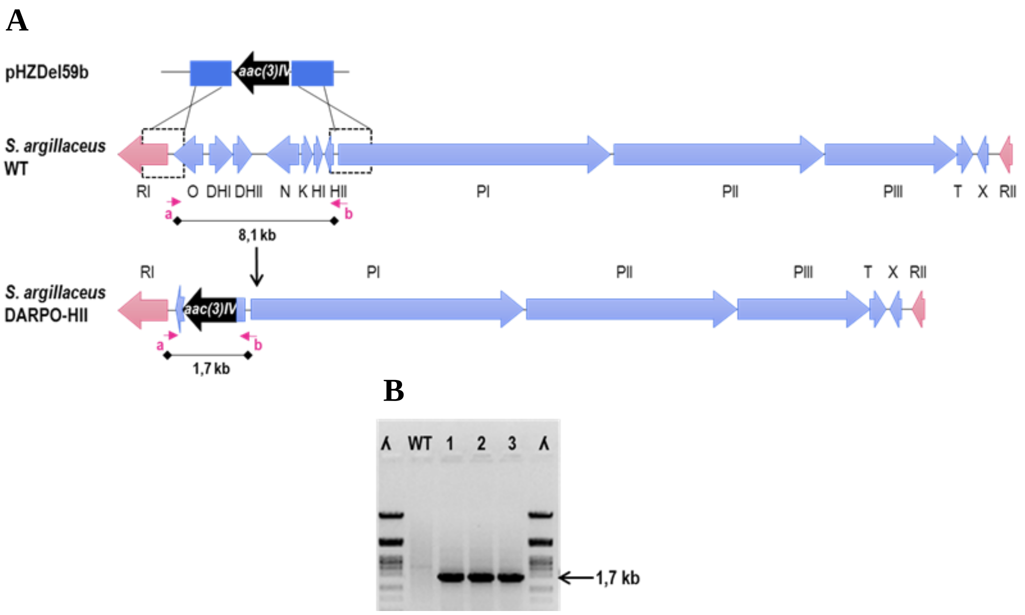

Figure S37: Comparison of gene organization of *arp* and PKS clusters in *S. argillaceus* and in *Streptomyces* sp. NRRL S-1022, respectively

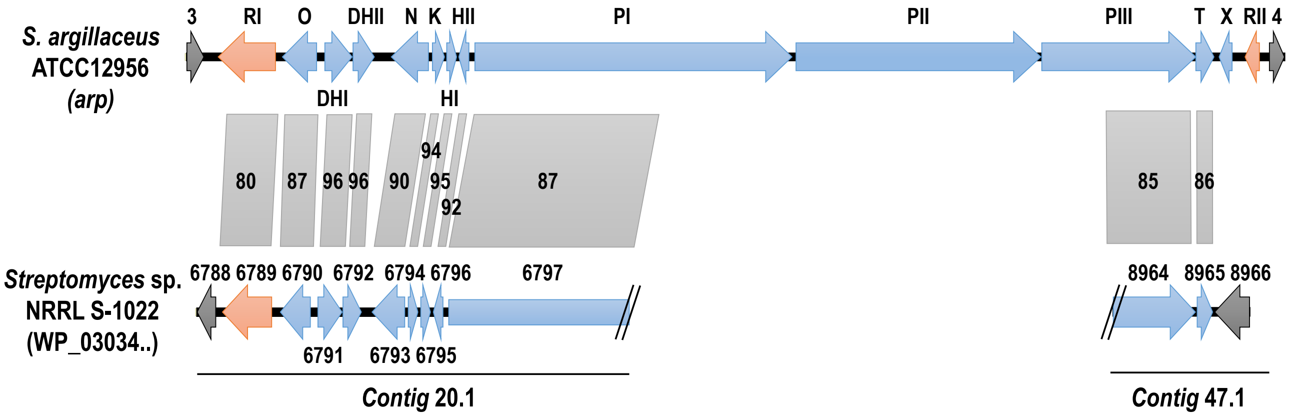

Supplement: Supplementary file 1 [file Presentation_1.PDF]
